# Hederagenin Attenuates Morphine Addiction via GABAA1‐Mediated Modulation of Ca^2^^+^–CaMK–CREB Signaling in Ventral Tegmental Dopaminergic Neurons

**DOI:** 10.1002/advs.77820

**Published:** 2026-09-27

**Authors:** Hongyang Jiang, Ziting Zhou, Nannan Xue, Jiasi Deng, Yuanyuan Zhou, Xinying Lv, Yuxuan Wang, Yi Zhang, Haotian Pan, Geyan Xu, Yinkun Tao, Mengran Li, Xin Cui, Hongyue Ma, Qian Wang, Zhigang Lu

**Affiliations:** ^1^ State Key Laboratory on Technologies for Chinese Medicine Pharmaceutical Process Control and Intelligent Manufacture Nanjing University of Chinese Medicine Nanjing China; ^2^ China Joint Graduate School of Traditional Chinese Medicine, Suzhou Jiangsu China; ^3^ School of Pharmacy Nanjing University of Chinese Medicine Nanjing China; ^4^ School of Integrative Medicine Nanjing University of Chinese Medicine Nanjing China; ^5^ Institute of Basic Research in Clinical Medicine China Academy of Chinese Medical Sciences Beijing China; ^6^ Jiangsu Collaborative Innovation Center of Chinese Medicinal Resources Industrialization Nanjing China; ^7^ Jiangsu Key Laboratory for High Technology Research of TCM Formulae Nanjing China; ^8^ School of International Education Nanjing University of Chinese Medicine Nanjing China; ^9^ Key Laboratory of Acupuncture and Medicine Research of Ministry of Education Nanjing University of Chinese Medicine Nanjing China

**Keywords:** GABA_A_ receptor α1 subunit, hederagenin, morphine‐conditioned place preference, RVG29‐modified liposomes, ventral tegmental area

## Abstract

Morphine‐associated contextual reward learning promotes maladaptive drug–context associations during opioid exposure. Hederagenin (HE), a bioactive compound from traditional Chinese medicine, has neuroprotective potential, but its role in contextual reward learning remains unclear. Here, we investigated the effects of HE on morphine‐induced conditioned place preference (CPP) acquisition and explored its target, downstream signaling, and brain‐delivery strategy. HE attenuated morphine CPP acquisition in both sexes and reduced established CPP expression. Chemical proteomics identified the GABAA receptor α1 subunit (GABAA1, encoded by GABRA1) as a prioritized HE‐associated target, supported by competitive pull‐down, non‐permeabilized HE‐biotin labeling, microscale thermophoresis, cellular thermal shift assay, and molecular docking. Mechanistically, HE reduced c‐Fos activation in ventral tegmental area (VTA) TH‐positive dopaminergic neurons, attenuated reward‐context‐associated VTA calcium responses, suppressed intracellular Ca^2^^+^ elevation in SH‐SY5Y cells, and reduced VTA CaMKII, PKA, and CREB phosphorylation. Animal‐level analyses linked CPP scores to compartment‐selective VTA calcium responses and p‐CREB/CREB ratios, whereas TH^+^/c‐Fos^+^ associations varied by treatment. Compared with non‐targeted liposomes, RVG29‐modified HE‐loaded liposomes showed greater brain‐ and VTA‐associated distribution and suppressed established CPP after a single administration. Together, these findings identify HE as a candidate modulator of morphine CPP through a GABAA1‐related VTA Ca^2^^+^–CaMK–cAMP–CREB mechanism and support RVG29 liposomes as an exploratory delivery strategy.

## Graphical abstract

Hederagenin is identified as a GABAA1‐targeting natural compound that suppresses morphine‐associated reward by restraining Ca^2^^+^–CaMK–cAMP–CREB signaling in ventral tegmental dopaminergic neurons. RVG29‐modified liposomal delivery further enhances brain accumulation and behavioral efficacy, providing a mechanistically defined strategy for natural‐product‐based intervention in opioid addiction.

## 1 Introduction

Opioid use disorder is a chronic, relapsing brain disorder characterized by compulsive drug use, persistent craving, and a high rate of relapse, posing a profound and long‐lasting threat to public health and socioeconomic systems [1]. Prolonged or repeated exposure to opioids such as morphine induces maladaptive remodeling of the central reward circuitry, whereby drug‐associated contexts and euphoric experiences are aberrantly consolidated into highly stable pathological memory traces, ultimately driving persistent drug‐seeking behavior and increasing vulnerability to relapse [2]. Although current opioid substitution therapies are effective in alleviating acute withdrawal symptoms, therapeutic strategies targeting the formation of psychological dependence and the stabilization of drug‐associated reward memory remain limited, underscoring the need to identify novel molecular targets within key neural circuits.

Dopaminergic neurons in the ventral tegmental area (VTA) represent a central hub for opioid‐induced reward processing and addiction‐related memory formation [3]. Accumulating evidence indicates that morphine induces long‐term synaptic and transcriptional adaptations by aberrantly activating intracellular second messenger systems, among which the cAMP/PKA/CREB signaling pathway plays a pivotal role in addiction‐related gene expression and behavioral reinforcement [4, 5]. Persistent upregulation of cAMP–CREB signaling has also been recognized as an important molecular basis for addiction‐related neuroplasticity and drug‐associated memory consolidation [6, 7]. As an essential upstream regulator of this pathway, intracellular Ca^2^^+^ signaling can convert transient electrophysiological changes into persistent molecular memory through activation of Ca^2^^+^/calmodulin‐dependent protein kinases (CaMKs). However, how Ca^2^^+^ signaling is precisely regulated in VTA dopaminergic neurons to drive pathological activation of the cAMP–CREB axis, as well as the key receptor‐mediated events and druggable nodes involved, remains insufficiently understood [8, 9, 10].

γ‐Aminobutyric acid (GABA)‐mediated inhibitory transmission plays a fundamental role in maintaining reward circuit homeostasis. As the principal inhibitory ligand‐gated chloride channels in the central nervous system, GABA_A_ receptors play a critical role in regulating neuronal excitability, voltage‐dependent calcium channel activity, and downstream Ca^2^^+^–dependent signaling. Among their subunits, the α1 subunit is a major determinant of receptor function and pharmacological properties. Enhanced GABAA1‐mediated inhibitory input effectively restricts Ca^2^^+^ influx, thereby indirectly modulating CaMK activity and cAMP production, providing a potential molecular bridge between inhibitory synaptic control and addiction‐related signaling networks [11, 12]. Despite increasing recognition of the role of GABAergic signaling in addiction, whether GABAA1 functions as an upstream integrative node of the Ca^2^^+^–CaMK–cAMP–CREB signaling axis and can be precisely targeted by small‐molecule compounds for opioid addiction intervention remains to be directly demonstrated.

**SCHEME 1:**
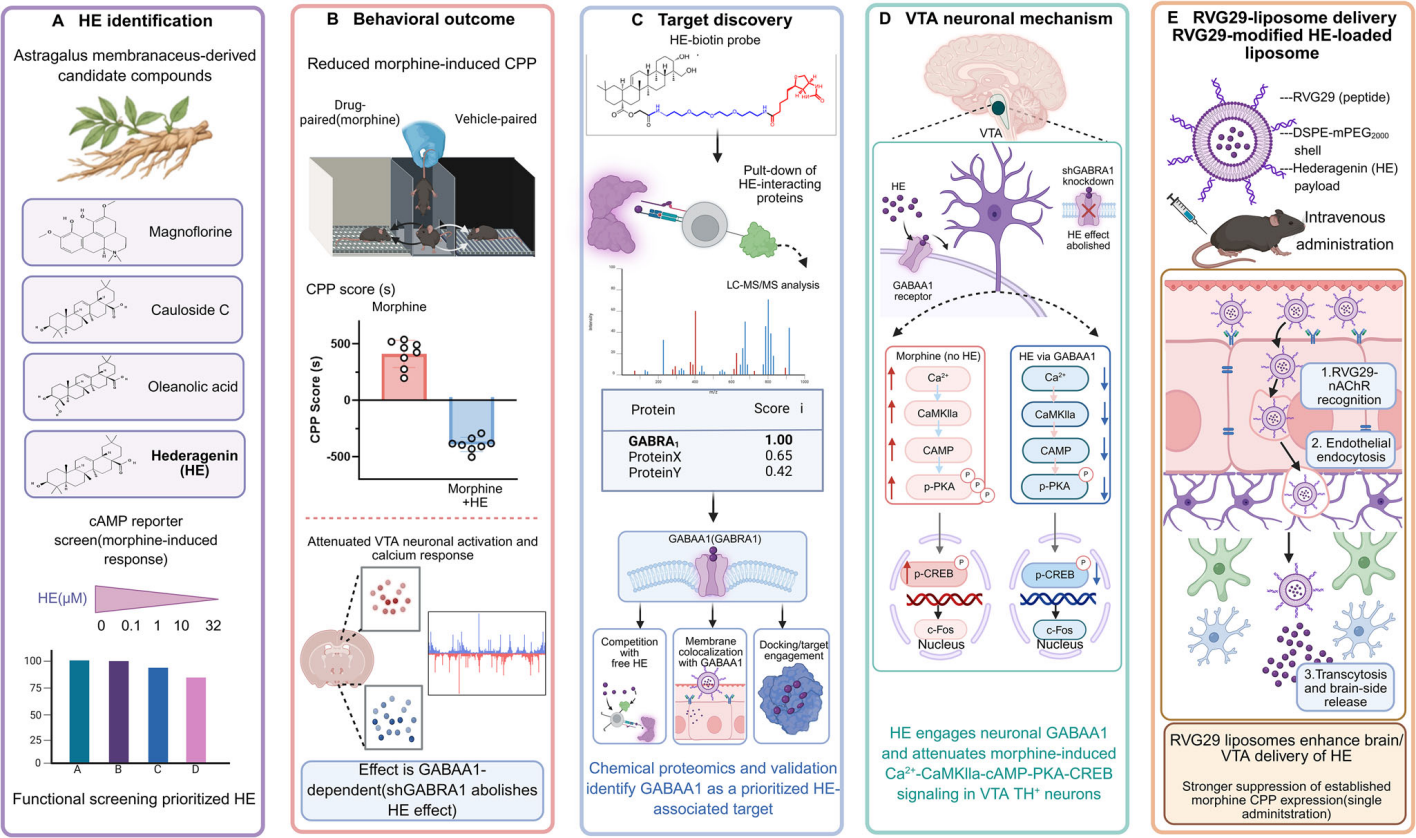
Proposed mechanism by which hederagenin attenuates morphine‐associated contextual reward learning through GABAA1‐related VTA Ca^2^^+^–CaMK–cAMP–CREB signaling and exploratory RVG29‐modified liposomes. The graphical abstract summarizes the main experimental logic and proposed mechanism of the study. (A) Active constituents derived from *Astragalus membranaceus* were functionally screened using a morphine‐induced cAMP reporter assay, leading to the prioritization of hederagenin (HE). (B) HE attenuated morphine‐induced conditioned place preference (CPP), reduced CPP scores, and decreased morphine‐associated VTA neuronal activation and calcium responses. (C) Chemical proteomics using an HE‐biotin probe, followed by LC–MS/MS analysis and target‐validation assays, identified the GABAA1 subunit, encoded by *GABRA1* and here referred to as GABAA1, as a prioritized HE‐associated candidate target. (D) Mechanistically, morphine increased VTA dopaminergic neuronal activity and activated Ca^2^^+^–CaMK–cAMP–CREB‐related signaling, whereas HE engagement with GABAA1 attenuated these signaling changes and reduced downstream CREB/c‐Fos activation. (E) As an exploratory delivery strategy, RVG29‐modified HE‐loaded liposomes were designed to enhance brain‐associated distribution. After intravenous administration, the RVG29‐modified formulation may interact with nAChR‐related recognition sites at the blood–brain barrier and undergo endothelial transcytosis, resulting in increased brain‐associated distribution of the liposomal formulation compared with non‐targeted liposomes. This schematic does not imply exclusive VTA delivery, intracellular HE release, or cell‐type‐specific targeting.

Natural products, owing to their structural diversity and multi‐target regulatory properties, represent a valuable source of therapeutic candidates for complex neuropsychiatric disorders. Hederagenin (HE), a pentacyclic triterpenoid found in several medicinal plants, has shown antidepressant‐like effects in preclinical rodent models [13]. However, whether HE modulates morphine‐associated contextual reward learning and the underlying molecular mechanisms remains unclear. Moreover, the role of HE in Ca^2^^+^‐dependent signaling regulation remains poorly characterized, and its limited blood–brain barrier permeability poses an additional challenge for central nervous system translation. Accordingly, the present study integrates chemical proteomics, molecular interaction validation, and cellular and behavioral analyses to systematically elucidate the molecular mechanism by which HE attenuates morphine‐associated contextual reward learning, uncovering its regulatory role in the Ca^2^^+^–CaMK–cAMP–CREB signaling axis within VTA dopaminergic neurons and providing a mechanistic framework for the targeted delivery and translational development of natural‐product‐based interventions for opioid‐associated behaviors (Scheme 1).

## 2 Results

### 2.1 Hederagenin Alleviates Morphine‐Induced cAMP Accumulation and the Acquisition of CPP in Mice

**FIGURE 1:**
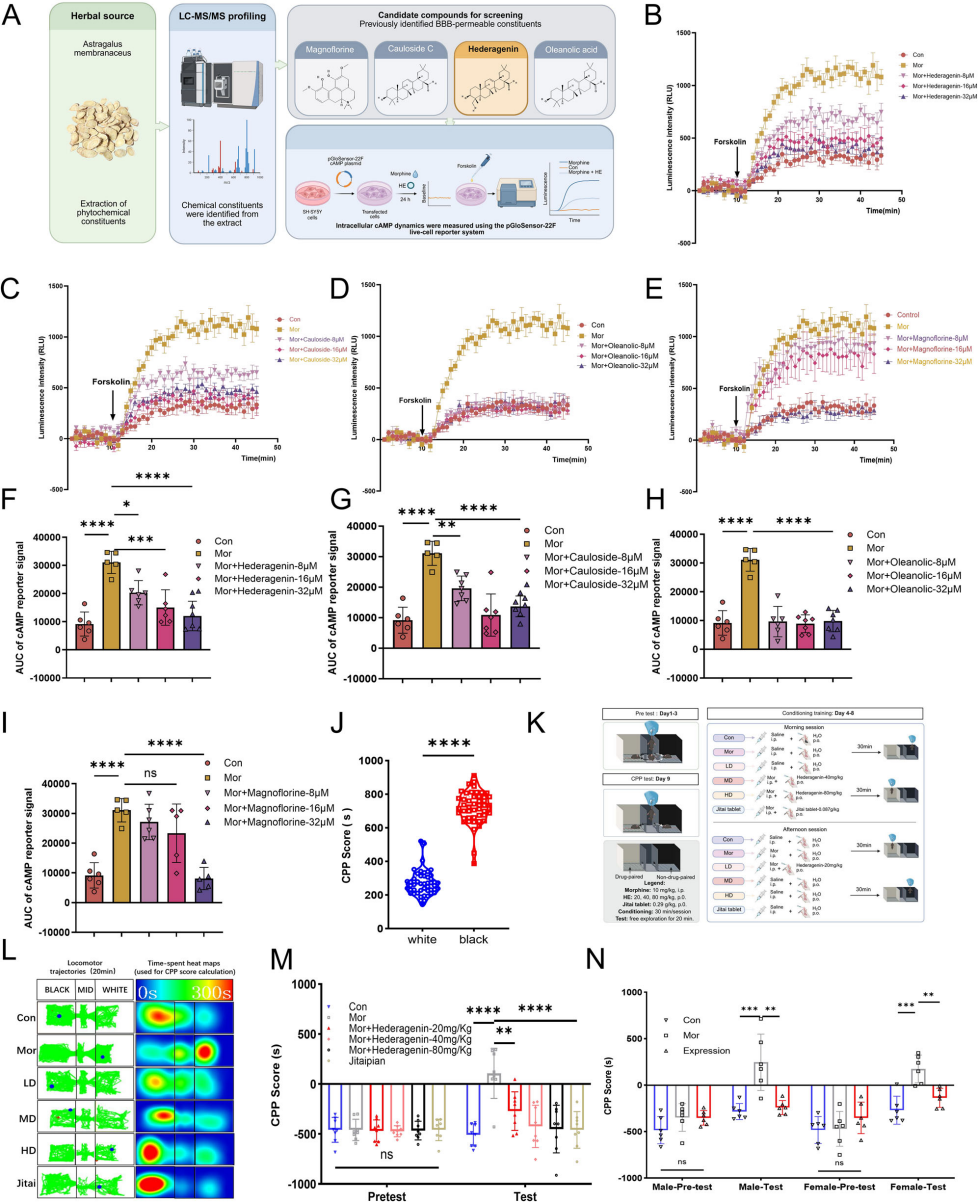
Hederagenin attenuates morphine‐induced cAMP elevation and conditioned place preference (CPP) acquisition in mice. (A) Schematic workflow for the identification and screening of blood–brain barrier‐permeable active constituents from *Astragalus membranaceus* and evaluation of their effects on morphine‐induced cAMP signaling in vitro. (B–E) Real‐time luminescence traces showing the effects of hederagenin (B), cauloside C (C), oleanolic acid (D), and magnoflorine (E) on morphine‐induced intracellular cAMP elevation in SH‐SY5Y cells. Forskolin was used to stimulate cAMP accumulation during recording. (F–I) Quantification of the area under the curve (*AUC*) of cAMP reporter signals corresponding to (B–E). (J) Baseline chamber preference of mice in the CPP apparatus. Mice exhibited a significant innate preference for the black compartment over the white compartment. (K) Schematic illustration of the CPP conditioning schedule. HE or Jitai tablets were administered 30 min before morphine (10 mg/kg, i.p.) on morphine‐paired conditioning sessions, and Jitai tablets were used as a positive control. (L) Representative locomotor trajectories during the 20‐min CPP test and representative heat maps of time spent in each compartment. (M) CPP scores at the pretest and test phases in control, morphine‐treated, hederagenin‐treated, and Jitai tablet‐treated mice. Jitai tablets were administered at 0.087 g/kg (p.o.) as a positive control. (N) Pretest and test CPP scores in male and female mice in the CPP expression experiment. Morphine CPP was first established, and HE was administered before the post‐test to evaluate its effect on the expression of established morphine‐associated contextual preference. In the expression group, HE was administered at 80 mg/kg, p.o., before the post‐test. For panels F–I, each cAMP AUC dataset was analyzed separately using ordinary one‐way ANOVA followed by Dunnett's multiple‐comparisons test, with the Mor group as the reference. Panels B‐E and F‐I share the same Con and Mor control datasets because the four candidate compounds were evaluated in parallel within the same experiment. For panel J, chamber preference was analyzed using a paired two‐tailed Student's *t*‐test. For panel M, the pretest and test data were analyzed separately using ordinary one‐way ANOVA followed by Dunnett's multiple‐comparisons test, with the Mor group as the reference. For panel N, the male and female pretest data and the female test data were analyzed separately using ordinary one‐way ANOVA followed by Dunnett's multiple‐comparisons test, with the Mor group as the reference; the male test data were analyzed using Welch's one‐way ANOVA followed by Dunnett's T3 multiple‐comparisons test, with the Mor group as the reference. Data are presented as mean ± SEM. Sample sizes and experimental units are specified for the corresponding panels. Complete inferential statistics, including test statistics, degrees of freedom, exact *p*‐values where available, effect‐size estimates, and multiplicity‐adjusted comparisons, are provided in Table S2. ns, not significant; *p < 0.05; **p < 0.01; ***p < 0.001; ****p < 0.0001. Asterisks denote multiplicity‐adjusted *p*‐values for the comparisons indicated by the brackets. Each data point represents one biological replicate.

In our previous study, four active constituents from *Astragalus membranaceus* (*A. membranaceus*)—magnoflorine, cauloside C, hederagenin, and oleanolic acid—were identified as blood–brain barrier‐permeable constituents (Figure 1A). We therefore evaluated the effects of these four compounds on morphine‐induced intracellular cAMP elevation in an in vitro model. For the initial comparative screening, all four candidate compounds were tested in parallel under the same concentration gradient of 8, 16, and 32 µm. As shown in Figure 1B–I, all four active ingredients significantly attenuated the morphine‐induced increase in intracellular cAMP levels (****p < 0.0001; ***p < 0.001; **p < 0.01; *p < 0.05). Significant overall treatment effects were detected for hederagenin [*F*(4, 24) = 16.46, *p* < 0.0001, *η* ^2^ = 0.733], cauloside [*F*(4, 27) = 19.24, *p* < 0.0001, *η* ^2^ = 0.740], oleanolic acid [*F*(4, 26) = 29.72, *p* < 0.0001, *η* ^2^ = 0.821], and magnoflorine [*F*(4, 22) = 16.98, *p* < 0.0001, *η* ^2^ = 0.755]. Subsequent Dunnett‐adjusted comparisons were performed using the Mor group as the reference, with the complete adjusted *p*‐values provided in Table S2. Among them, hederagenin exhibited the clearest dose‐dependent inhibitory effect, whereas the other three compounds showed less obvious dose‐dependent patterns. These findings suggested that hederagenin exerted the most consistent inhibitory effect on morphine‐induced cAMP hyperactivation. To further determine whether this effect was behaviorally relevant, we next examined the effect of hederagenin on morphine‐induced conditioned place preference (CPP) acquisition in mice. The CPP procedure consisted of three phases: baseline, conditioning, and testing, and a schematic illustration of the CPP conditioning schedule is shown in Figure 1K. During the baseline phase, the natural chamber preference of all mice was recorded for subsequent group allocation. As shown in Figure 1J, mice spent significantly more time in the black compartment than in the white compartment (****p < 0.0001), indicating a marked innate preference for the black compartment [paired two‐tailed Student's *t*‐test, *t*(47) = 19.05, *p* < 0.0001, Cohen's *d*_z_ = 2.75; mean paired difference = 449.6 s, 95% CI, 402.1–497.1]. Therefore, in this biased CPP design, the white compartment was designated as the drug‐paired chamber and the black compartment as the non‐drug‐paired chamber. No significant overall group difference in CPP scores was detected during the pretest phase [*F*(5, 42) = 0.03, *p* = 0.9996, *η* ^2^ = 0.003; Figure 1M, Table S2]. After 5 days of conditioning, the CPP test was performed. To provide a pharmacological reference in the morphine‐induced CPP paradigm, Jitai tablets were included as a positive control. The CPP results showed that compared with the control group, morphine‐treated mice showed a significantly increased preference for the drug‐paired compartment (Figure 1M), indicating that the morphine‐induced CPP mouse model was successfully established. Compared with the model group, CPP scores were significantly reduced in the Jitai tablet group, indicating that Jitai tablet treatment attenuated morphine‐induced CPP acquisition (Figure 1M). Compared with the model group, CPP scores were significantly reduced in all hederagenin‐treated groups (Figure 1M; ****p < 0.0001; **p < 0.01). The overall treatment effect at test was significant [*F*(5, 42) = 10.28, *p* < 0.0001, *η* ^2^ = 0.550; Table S2]. Dunnett‐adjusted comparisons showed that morphine significantly increased CPP scores relative to the Con group (adjusted *p* < 0.0001), whereas CPP scores were reduced relative to the Mor group in the HE‐LD group (adjusted *p* = 0.0022) and in the HE‐MD and HE‐HD groups (both adjusted *p* < 0.0001). Jitai treatment also reduced CPP scores relative to the Mor group (adjusted *p* < 0.0001). These results indicated that different doses of hederagenin significantly attenuated morphine‐induced CPP acquisition. Representative trajectories and heat maps for each group are shown in Figure 1L. Morphine conditioning induced robust CPP acquisition, whereas HE treatment reduced morphine‐induced CPP scores. Because the mechanistic experiments in this study were designed around the acquisition phase, subsequent analyses focused on this acquisition‐related CPP model. To address whether HE also affects established morphine‐associated contextual preference, HE was administered before the post‐test after CPP had been established, and HE reduced the expression of established morphine CPP (Figure 1N; **p < 0.01). No significant overall group differences were detected at pretest in male [*F*(2, 15) = 2.16, *p* = 0.1503, *η* ^2^ = 0.223] or female mice [*F*(2, 15) = 1.11, *p* = 0.3562, *η* ^2^ = 0.129]. Significant overall group effects were detected at test in male mice [Welch's *W*(2, 9.645) = 13.40, *p* = 0.0016, *η* ^2^ (descriptive) = 0.664] and female mice [*F*(2, 15) = 16.84, *p* = 0.0001, *η* ^2^ = 0.692; Table S2]. Raw compartment occupancy analysis further confirmed that changes in CPP scores were not attributable to increased neutral‐compartment occupancy (Figure S1). Additional validation experiments showed that HE attenuated morphine‐induced CPP acquisition in both male and female mice and under matched conditioning‐time conditions, supporting that its effect was not restricted to sex or time‐of‐day differences (Figure S2). No significant overall group differences were detected at pretest in either sex. At test, significant overall group effects were detected in both male [*F*(3, 20) = 13.59, *p* < 0.0001, *η* ^2^ = 0.671] and female cohorts [*F*(3, 20) = 12.47, *p* < 0.0001, *η* ^2^ = 0.652; Figure S2A, Table S2].

### 2.2 Hederagenin Does Not Cause Major Behavioral Confounds in Mice

**FIGURE 2:**
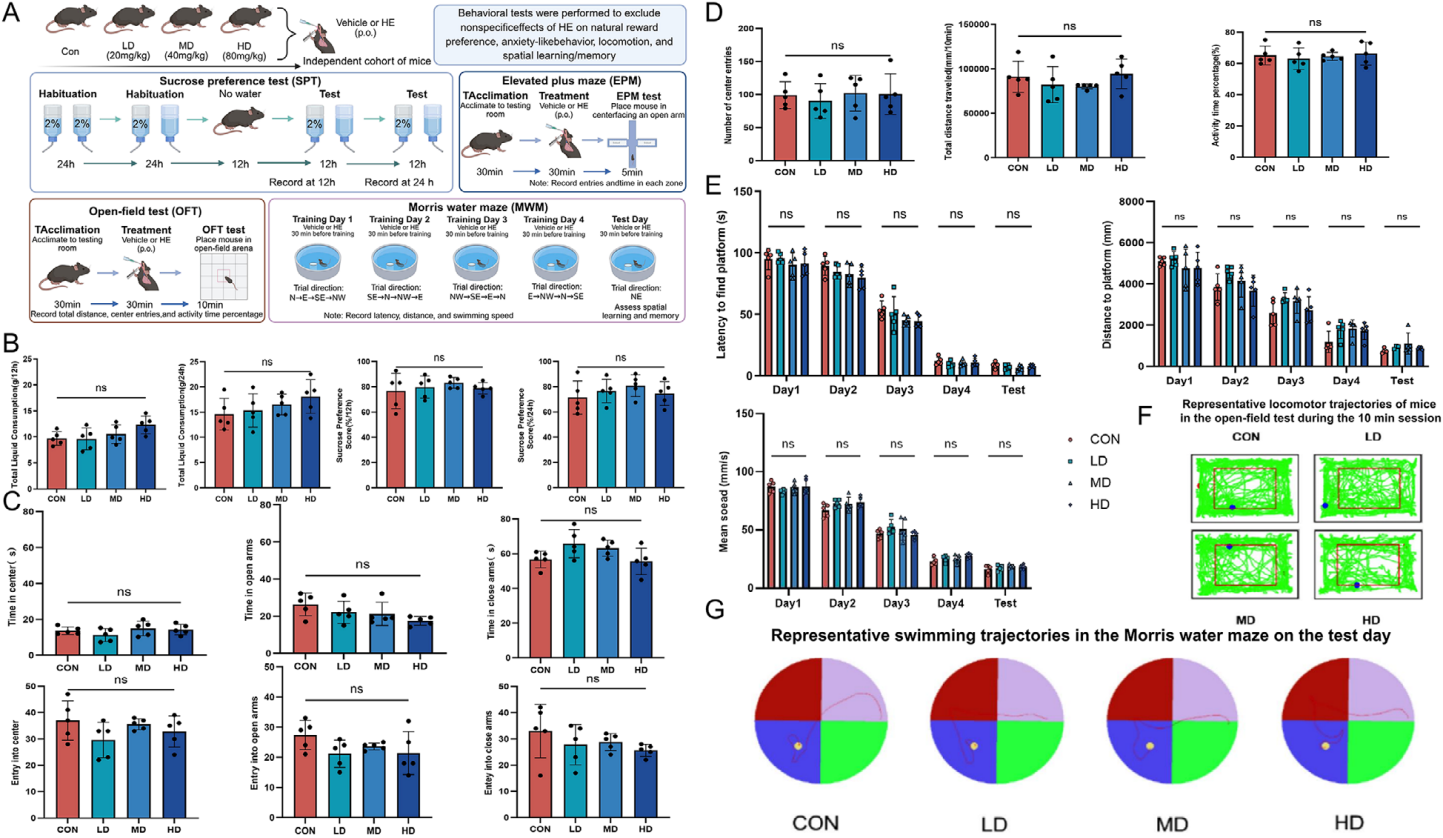
Hederagenin does not cause major behavioral confounds in mice. (A) Schematic overview of the behavioral assays used to evaluate the potential nonspecific effects of HE alone. (B) Sucrose preference test: total fluid intake (12, 24 h) and sucrose preference (%) (12, 24 h). (C) Elevated plus maze: time spent in the center zone, open arms, and closed arms, and the corresponding number of entries into each zone. (D) Open‐field test: center entries, total distance traveled, and activity time percentage. (E) Morris water maze: latency to find the platform, distance to the platform, and swimming speed. (F) Representative locomotor trajectories in the open‐field test during the 10‐min session. (G) Representative swimming trajectories in the Morris water maze on the test day. Mice were randomly assigned to Con, LD (20 mg/kg), MD (40 mg/kg), and HD (80 mg/kg) groups. For panel B, data were analyzed using ordinary one‐way ANOVA followed by Tukey's multiple‐comparisons test. For panel C, center‐zone time, open‐arm time, closed‐arm time, and center‐zone entries were analyzed using ordinary one‐way ANOVA followed by Tukey's test; open‐arm and closed‐arm entries were analyzed using Welch's one‐way ANOVA followed by Dunnett's T3 test, with the Con group as the reference. For panel D, center entries and activity time were analyzed using ordinary one‐way ANOVA followed by Tukey's test, whereas total distance was analyzed using Welch's one‐way ANOVA followed by Dunnett's T3 test. For panel E, each training day and the test phase were analyzed separately. Latency and swimming‐speed data were analyzed using ordinary one‐way ANOVA followed by Tukey's test at each time point. For distance, Day 1 was analyzed using Welch's one‐way ANOVA, whereas Days 2–4 and the test phase were analyzed using ordinary one‐way ANOVA followed by Tukey's test. Data are presented as mean ± SEM. Sample sizes and the experimental unit are specified for the corresponding panels. Complete inferential statistics, including test statistics, degrees of freedom, exact *P*‐values, effect‐size estimates, and multiplicity‐adjusted comparisons, are provided in Table S2. ns, not significant; *p < 0.05; **p < 0.01; ***p < 0.001; ****p < 0.0001. Asterisks denote multiplicity‐adjusted *p*‐values for the comparisons indicated by the brackets. Each data point represents one mouse.

To exclude the possibility that the inhibitory effect of HE on morphine‐CPP was secondary to nonspecific behavioral alterations, we further evaluated the effects of HE alone on natural reward preference, anxiety‐like behavior, locomotor activity, and spatial learning/memory in an independent cohort of mice using a series of behavioral assays, including the sucrose preference test (SPT), elevated plus maze (EPM), open‐field test (OFT), and Morris water maze (MWM) (Figure 2A). Natural reward preference was first assessed by the SPT. No significant differences were observed among the Con, LD, MD, and HD groups in total fluid intake or sucrose preference (%) at either 12 or 24 h (Figure 2B), indicating that HE did not significantly affect natural reward preference under the present experimental conditions.

We next examined anxiety‐like behavior using the EPM. Compared with the Con group, HE treatment did not significantly alter the time spent in the center zone, open arms, or closed arms, nor did it significantly affect the number of entries into these regions (Figure 2C), suggesting that HE did not induce obvious anxiety‐related behavioral changes at the tested doses. To further determine whether HE affected spontaneous locomotor activity and exploratory behavior, mice were subjected to the OFT. HE treatment did not significantly alter the number of entries into the center zone, the total distance traveled during the 10‐min session, or the activity time percentage (Figure 2D). Representative locomotor trajectories are shown in Figure 2F, illustrating that HE did not produce obvious changes in the overall exploration pattern of mice in the open field. To determine whether HE affected spatial learning and memory, mice were further tested in the MWM. No significant group differences were detected in latency to find the platform, distance to the platform, or swimming speed on each training day and during the test phase (Figure 2E), suggesting that HE did not impair spatial learning or memory under the present experimental conditions. Representative swimming trajectories on the test day are shown in Figure 2G. No significant omnibus group effects were detected across the sucrose‐preference, elevated‐plus‐maze, open‐field, or Morris‐water‐maze outcomes (all *p* > 0.05; complete inferential statistics are provided in Table S2).

Taken together, these results indicate that HE alone does not induce major nonspecific alterations in reward‐related, anxiety‐related, locomotor, or cognitive behaviors. Thus, the inhibitory effect of HE on morphine CPP is unlikely to be secondary to changes in natural reward preference, anxiety‐like behavior, locomotion, or cognitive performance.

### 2.3 Hederagenin Attenuates Morphine‐Induced Activation of TH‐Positive VTA Dopaminergic Neurons and Reward‐Context Calcium Responses During CPP

**FIGURE 3:**
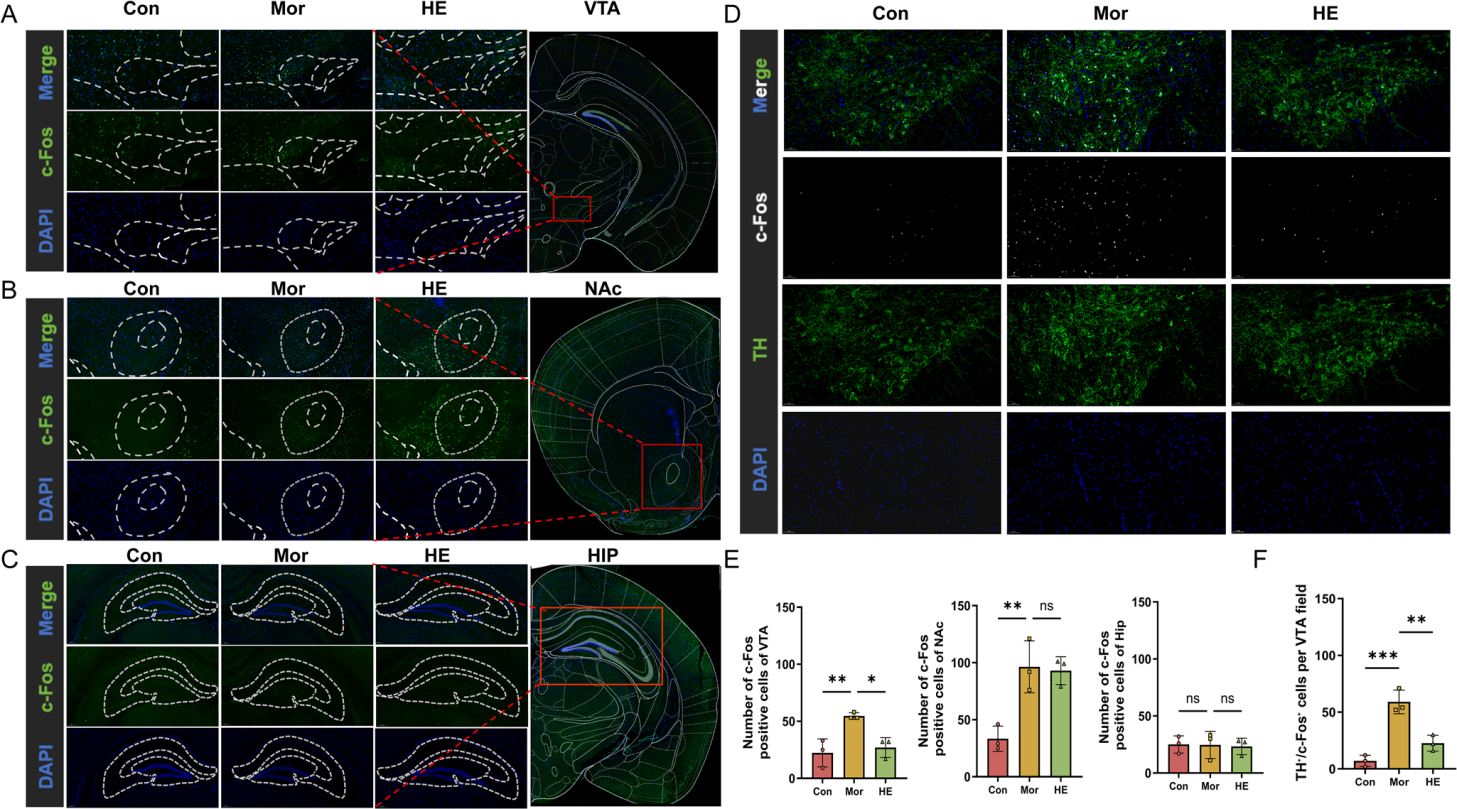
HE attenuates morphine‐induced c‐Fos activation in TH‐positive VTA dopaminergic neurons. (A–C) Representative immunofluorescence images of c‐Fos staining in the VTA (A), NAc (B), and Hip (C) after CPP acquisition. DAPI labels nuclei, and c‐Fos was used as an activity‐related marker. The outlined regions indicate the analyzed anatomical areas. (D) Representative TH/c‐Fos double immunofluorescence images in the VTA. TH‐positive dopaminergic neurons are shown in green, c‐Fos‐positive nuclei in white, and DAPI in blue. (E) Quantification of c‐Fos‐positive cells in the VTA, NAc, and Hip. The same *y*‐axis scale was used for all brain‐region bar graphs. (F) Quantification of TH^+^/c‐Fos^+^ double‐positive cells per VTA field. Data are presented as mean ± SEM, *n* = 3 mice per group. Each data point represents one mouse. The VTA, NAc, and Hip c‐Fos‐positive cell counts in panel E were analyzed separately using ordinary one‐way ANOVA followed by Dunnett's multiple‐comparisons test, with the Mor group as the reference. The VTA TH^+^/c‐Fos^+^ double‐positive cell counts in panel F were analyzed using ordinary one‐way ANOVA followed by Dunnett's multiple‐comparisons test, with the Mor group as the reference. ns, not significant; *p < 0.05; **p < 0.01; ***p < 0.001. Con, control group; Mor, morphine‐treated group; HE, hederagenin‐treated group. Scale bars, 50 µm in (A–D).

To evaluate region‐specific c‐Fos activation during morphine‐induced CPP acquisition, c‐Fos immunofluorescence was first examined in addiction‐related brain regions, including the ventral tegmental area (VTA), nucleus accumbens (NAc), and hippocampus (Hip). Representative images showed increased c‐Fos signals in the VTA and NAc after morphine conditioning, whereas the Hip displayed relatively limited changes (Figure 3A–C). Quantitative analysis further showed that morphine markedly increased the number of c‐Fos‐positive cells in the VTA and NAc compared with the control group. HE treatment significantly reduced morphine‐induced c‐Fos activation in the VTA, whereas no significant reduction was observed in the NAc. In contrast, c‐Fos‐positive cell counts in the Hip were not significantly different among groups (Figure 3E; **p < 0.01; *p < 0.05). Significant overall group effects were detected for c‐Fos‐positive cell counts in the VTA [*F*(2, 6) = 11.82, *p* = 0.0083, *η* ^2^ = 0.798] and NAc [*F*(2, 6) = 14.27, *p* = 0.0052, *η* ^2^ = 0.826], but not in the hippocampus [*F*(2, 6) = 0.03, *p* = 0.9727, *η* ^2^ = 0.009; Table S2]. Because c‐Fos/DAPI staining alone does not define the cellular identity of activated cells, TH/c‐Fos double immunofluorescence staining was further performed in the VTA. TH was used to identify dopaminergic neurons, whereas c‐Fos was used as an activity‐related marker. Morphine markedly increased the number of TH^+^/c‐Fos^+^ double‐positive cells in the VTA, indicating enhanced activation of TH‐positive VTA dopaminergic neurons during CPP acquisition. HE treatment significantly reduced the number of TH^+^/c‐Fos^+^ cells compared with the morphine group (Figure 3D,F; ***p < 0.001; *p < 0.05). The overall group effect on VTA TH^+^/c‐Fos^+^ double‐positive cell counts was significant [*F*(2, 6) = 34.93, *p* = 0.0005, *η* ^2^ = 0.921; Table S2]. These results indicate that HE attenuates morphine‐induced c‐Fos activation in TH‐positive VTA dopaminergic neurons.

**FIGURE 4:**
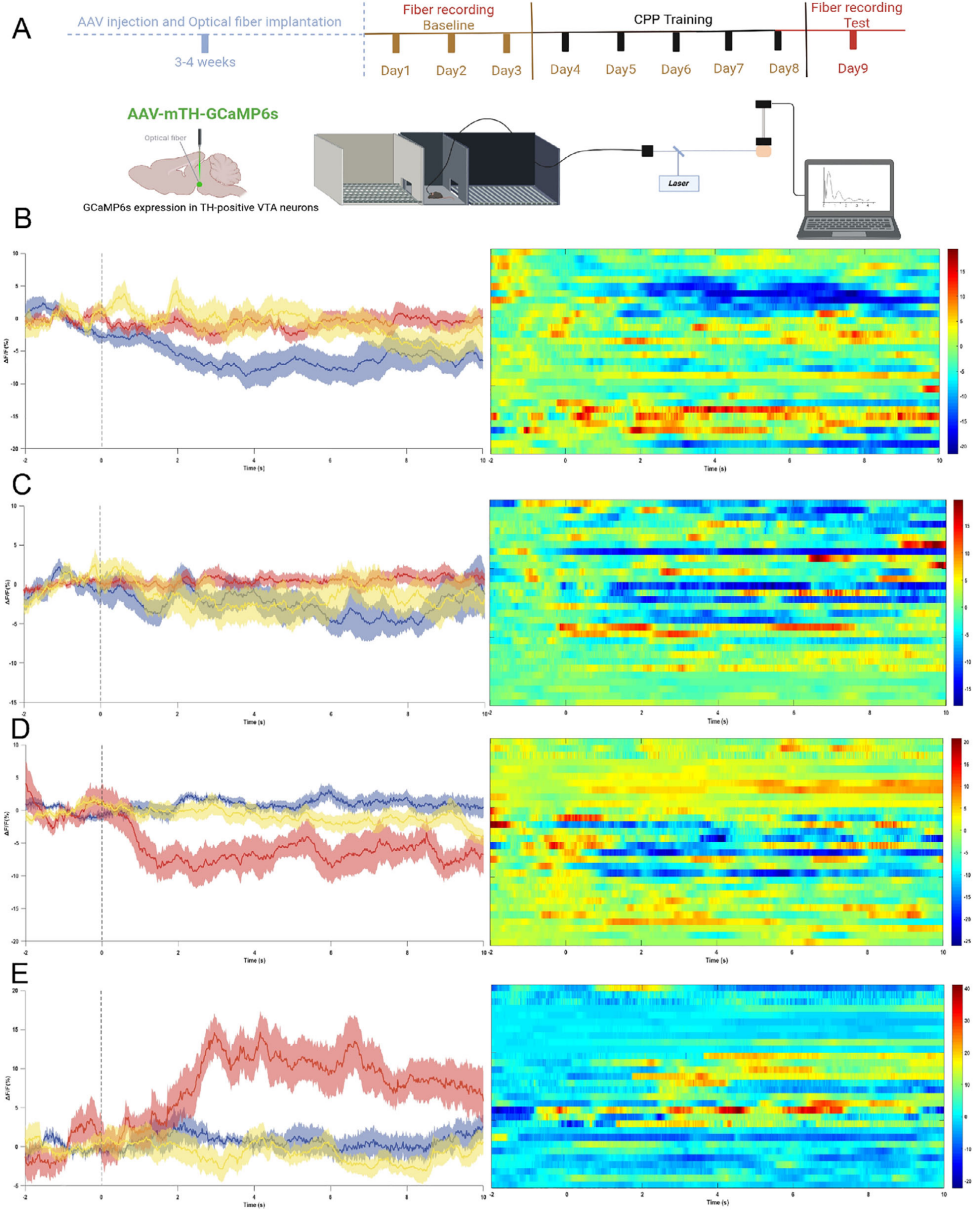
HE attenuates morphine‐induced reward‐context calcium responses in VTA dopaminergic neurons during CPP. (A) Schematic illustration of AAV injection, optical fiber implantation, CPP training schedule, and in vivo fiber photometry recording of calcium activity in VTA dopaminergic neurons. AAV‐mTH‐G6S was injected into the VTA to express GCaMP6s under the control of the mTH promoter, enabling calcium signal recording preferentially in TH‐positive dopaminergic neurons. (B,C) Calcium responses during the pretest session upon entry into the non‐drug‐paired compartment (B) and drug‐paired compartment (C). Average Δ*F*/*F* traces and heat maps are shown. (D,E) Calcium responses during the CPP test session upon entry into the non‐drug‐paired compartment (D) and drug‐paired compartment (E). Morphine conditioning enhanced VTA calcium activity upon entry into the drug‐paired compartment, whereas HE treatment attenuated this reward‐context‐associated calcium response. Fiber‐photometry data were obtained from the Con, Mor, and Mor + HE (80 mg/kg) groups (*n* = 3 mice per group). Data are presented as mean ± SEM.

To further assess reward‐context‐related VTA neuronal activity during CPP behavior, in vivo fiber photometry was performed using AAV‐mTH‐G6S to monitor calcium dynamics in VTA TH‐promoter‐targeted dopaminergic neurons (Figure 4A). In this construct, the mTH promoter drives GCaMP6s expression preferentially in TH‐positive dopaminergic neurons. During the pretest session, calcium responses did not differ markedly among groups upon entry into either the non‐drug‐paired or drug‐paired compartment, indicating comparable baseline activity before conditioning (Figure 4B,C).

After morphine conditioning, morphine‐treated mice exhibited an increased VTA calcium response upon entry into the drug‐paired compartment, whereas no comparable enhancement was observed in the non‐drug‐paired compartment (Figure 4D,E). HE treatment attenuated this morphine‐induced reward‐context‐associated calcium response, as reflected by reduced Δ*F*/*F* signals and weaker event‐related calcium activity in the drug‐paired compartment (Figure 4E).

Together, these histological and functional data suggest that morphine‐induced CPP is associated with increased c‐Fos activation in TH‐positive VTA dopaminergic neurons and enhanced reward‐context‐associated VTA calcium responses. HE attenuated both TH^+^/c‐Fos^+^ activation and reward‐context‐associated calcium responses, supporting a modulatory effect of HE on VTA dopaminergic neuronal activity during morphine‐induced CPP. When all matched animals were pooled, CPP scores were positively correlated with VTA TH^+^/c‐Fos^+^ double‐positive cell counts (two‐sided Pearson's *r* = 0.661, *p* = 0.0073, *n* = 15; Figure S4A). However, treatment‐stratified analyses showed negative correlations in the Con group (*r* = −0.992, *p* = 0.0009) and Mor + HE group (*r* = −0.989, *p* = 0.0014), but a positive correlation in the Mor group (*r* = 0.997, *p* = 0.0002; *n* = 5 per group; Figure S4B). The CPP score × treatment group interaction was significant [*F*(2, 9) = 314.24, *p* < 0.0001, partial *η* ^2^ = 0.986], indicating that the direction and regression slope of the relationship differed across treatment groups. Therefore, the pooled positive association should not be interpreted as a uniform continuous relationship across all treatment conditions. To examine the association between CPP scores and compartment‐selective VTA calcium activity, we analyzed a matched fiber‐photometry subset of the CPP cohort in which behavioral and calcium data were obtained from the same mice (Con, Mor, and Mor + HE [HD, 80 mg/kg]; *n* = 3 mice per group; *n* = 9 in total). ΔAUC was defined as the 0–10 s post‐entry AUC in the drug‐paired compartment minus that in the non‐drug‐paired compartment. In the pooled matched dataset, a significant positive correlation was observed between CPP scores and Δ*AUC* (two‐sided Pearson's *r* = 0.883, *p* = 0.0016; Figure S5A). Group‐specific regression slopes were positive in all three groups (Figure S5B), but were interpreted descriptively because of the limited within‐group sample size.

### 2.4 Chemical Proteomics Identifies GABAA1 as a Candidate Target of Hederagenin in Morphine Addiction

**FIGURE 5:**
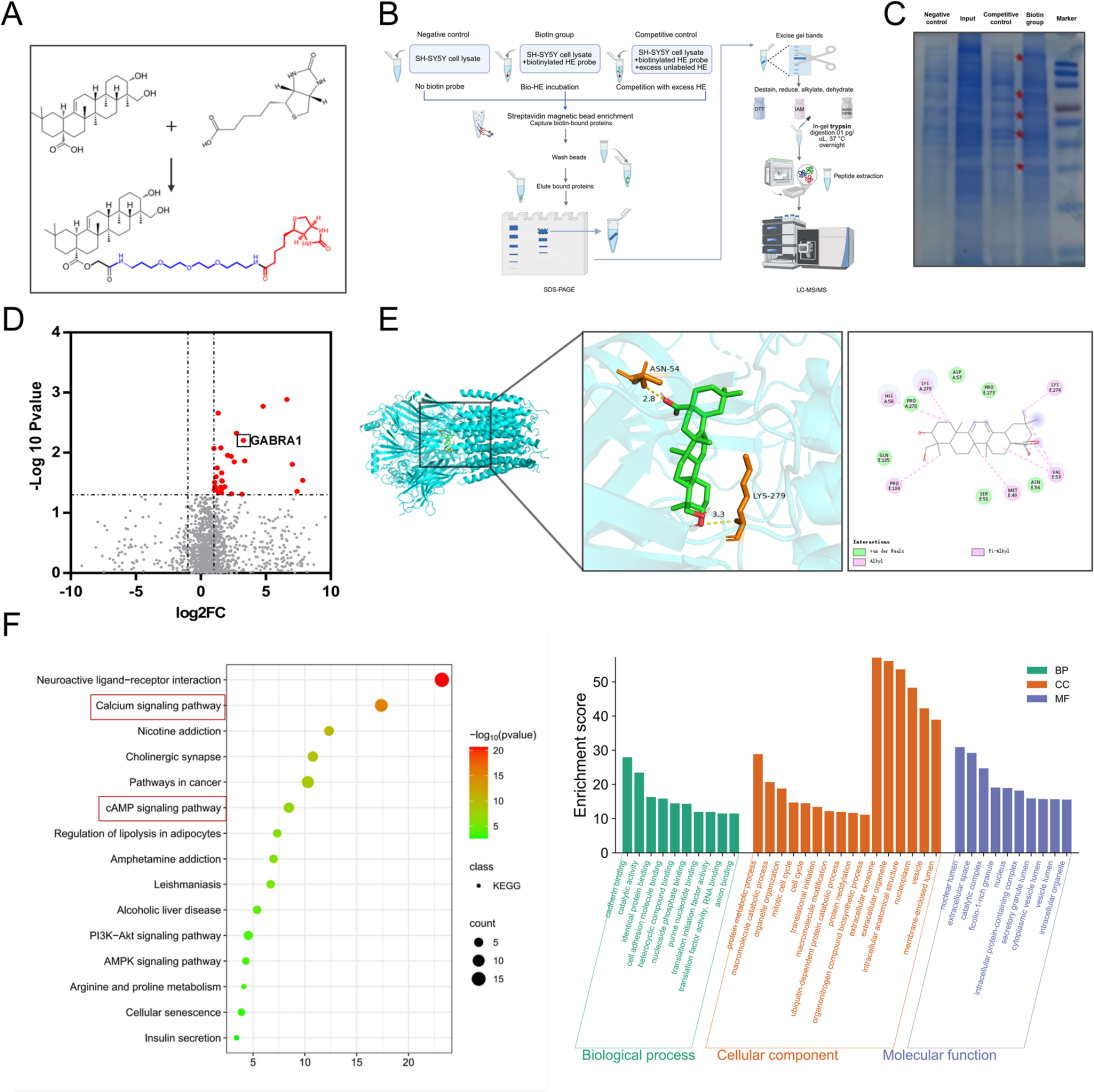
Chemical proteomic analysis and target prioritization of hederagenin. (A) Synthetic route of the biotin‐labeled hederagenin (HE‐biotin) probe. (B) Schematic workflow for target identification by chemical proteomics, including negative control, biotin group, and competitive control, followed by streptavidin magnetic bead enrichment, SDS–PAGE, in‐gel tryptic digestion, and LC–MS/MS analysis. (C) Representative SDS–PAGE image of enriched proteins from the different groups. Red asterisks indicate bands showing differential enrichment relative to both the negative control and competitive control groups. (D) Volcano plot showing the distribution of filtered candidate proteins after exclusion of negative‐control background and differential comparison between the biotin group and the competitive control group; the prioritized candidate is highlighted. (E) Molecular docking model illustrating a plausible interaction between HE and the experimentally resolved human *α*1*β*3*γ*2L GABAA receptor (PDB ID: 6HUG) in the selected extracellular *α*1(+)/*γ*2(−) pocket, together with the predicted local interaction pattern. Diazepam was used as a control ligand for basic validation of the docking setup. (F) GO and KEGG enrichment analyses of the filtered candidate protein set.

Our preliminary in vitro and behavioral studies showed that hederagenin (HE) significantly attenuated morphine‐induced CPP and inhibited abnormal cAMP signaling, but its direct molecular target remained unclear. Therefore, we synthesized a biotinylated HE probe and performed chemical proteomics analysis in SH‐SY5Y cell lysates to identify potential HE‐associated proteins (Figure 5A). The overall workflow of biotin labeling and protein target identification is shown in Figure 5B.

As shown in Figure 5C, compared with the negative control and competitive control groups, the biotin group displayed multiple enriched protein bands, indicating effective and specific labeling of HE‐associated proteins. After excluding nonspecific background proteins detected in the negative control, differential proteomic analysis of the biotin group versus the competitive control group identified a filtered set of candidate proteins, in which GABAA1 was highlighted as a prioritized candidate target (Figure 5D). Given that previous studies have confirmed an important role for GABAA1 in reward circuitry and dopaminergic neuronal excitability [14], GABAA1 was selected for further investigation. Furthermore, GO and KEGG enrichment analyses of the filtered candidate proteins showed significant enrichment in pathways related to calcium and cAMP signaling (Figure 5F), providing functional support for the involvement of these candidates in addiction‐related signaling regulation. Molecular docking analysis further supported a plausible interaction between HE and GABAA1, with the best‐scoring docking pose showing a predicted binding energy of −10 kcal/mol (Figure 5E). The docking model also suggested multiple predicted interacting residues in the selected pocket, with van der Waals, alkyl, and *π*–alkyl interactions predominating. Together, these results suggested that GABAA1 may serve as a candidate target of HE involved in the regulation of addiction‐related signaling pathways. Given that GABAA1 activation can reduce neuronal excitability by limiting calcium influx and that intracellular Ca^2^^+^ is an important upstream regulator of cAMP signaling, these findings suggest that HE may attenuate morphine‐induced CPP, at least in part, through GABAA1‐associated regulation of Ca^2^^+^‐ and cAMP‐related signaling pathways [15].

### 2.5 GABAA1 is Required for HE‐Mediated Suppression of Morphine‐Induced Neuronal Activation and CPP

**FIGURE 6:**
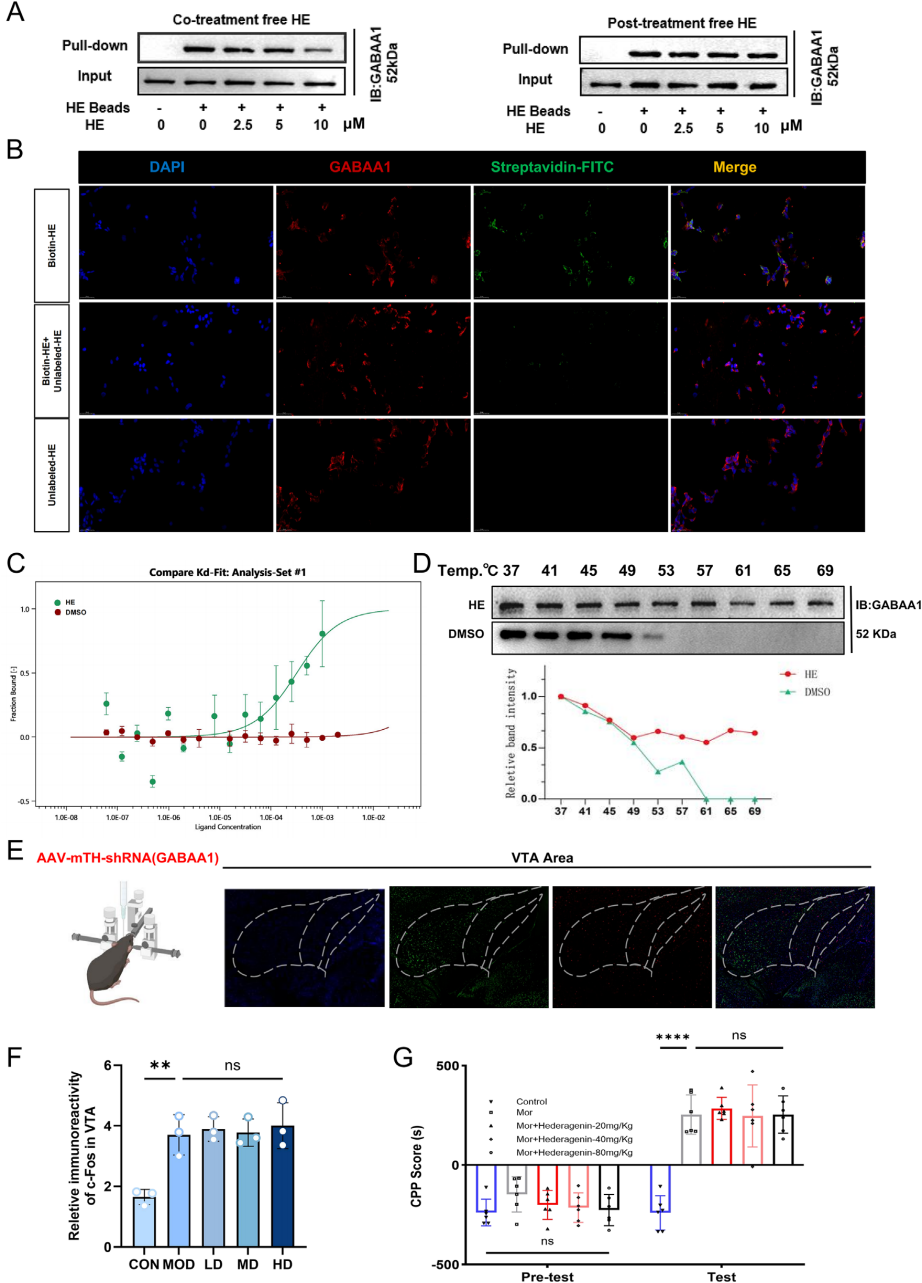
GABAA1 is required for HE‐mediated suppression of morphine‐induced neuronal activation and CPP. (A) Competitive pull‐down analysis of HE‐biotin‐associated GABAA1 enrichment in SH‐SY5Y cell lysates. Free unlabeled HE was applied at 0, 2.5, 5, and 10 µm under co‐treatment and post‐treatment competition conditions, followed by immunoblotting for GABAA1. (B) Non‐permeabilized immunofluorescence staining of SH‐SY5Y cells showing DAPI (blue), GABAA1 immunoreactivity (red), streptavidin‐FITC detection of HE‐biotin labeling (green), and merged images. HE‐biotin produced surface‐associated labeling that overlapped with GABAA1 immunoreactivity, whereas excess unlabeled HE markedly reduced the streptavidin‐FITC signal, supporting competitive inhibition of HE‐biotin labeling by the parent compound. Scale bar, 50 µm. (C) Microscale thermophoresis (MST) analysis showing concentration‐dependent binding between HE and GABAA1, with an apparent dissociation constant (*K*_d_) of 0.28 µm. (D) Cellular thermal shift assay (CETSA) showing increased thermal stability of GABAA1 in the presence of HE compared with the DMSO control. (E) Schematic and representative immunofluorescence images showing stereotaxic delivery of AAV‐mTH‐shGABAA1 into the ventral tegmental area (VTA). The mTH promoter was used to preferentially target TH‐positive dopaminergic neurons, and the shRNA cassette was designed to reduce *GABRA1*/GABAA1 expression in the VTA. Immunofluorescence images show DAPI (blue), TH‐positive dopaminergic neurons (green), GABAA1 (red), and merged signals in the VTA region. Scale bar: 50 µm. (F) Quantification of c‐Fos immunoreactivity in the VTA after AAV‐mTH‐shGABAA1‐mediated knockdown in all groups. (G) CPP scores after AAV‐mTH‐shGABAA1‐mediated knockdown in all groups. Morphine‐induced CPP remained detectable, whereas HE no longer suppressed CPP under GABAA1 knockdown conditions. Groups in (F) and (G) were Con, Mor, Mor + HE‐LD, Mor + HE‐MD, and Mor + HE‐HD. LD, MD, and HD indicate HE at 20, 40, and 80 mg/kg, respectively. Data are presented as mean ± SEM. Panel F was analyzed using ordinary one‐way ANOVA followed by Dunnett's multiple‐comparisons test, with the Mor group as the reference. For panel G, pretest and test CPP scores were analyzed separately using ordinary one‐way ANOVA followed by Dunnett's multiple‐comparisons test, with the Mor group as the reference. Sample sizes and the experimental unit are specified for the corresponding panels. Complete inferential statistics, including test statistics, degrees of freedom, exact *P*‐values, effect‐size estimates, and multiplicity‐adjusted comparisons, are provided in Table S2. ns, not significant; *p < 0.05; **p < 0.01; ***p < 0.001; ****p < 0.0001. Asterisks denote multiplicity‐adjusted *p*‐values for the comparisons indicated by the brackets. Each data point represents one biological replicate.

To validate the interaction between hederagenin (HE) and GABAA1, competitive pull‐down assays were performed in SH‐SY5Y cell lysates using increasing concentrations of free unlabeled HE. As shown in Figure 6A, free HE at 0, 2.5, 5, and 10 µm was applied under both co‐treatment and post‐treatment conditions. In the co‐treatment assay, free HE dose‐dependently reduced HE‐biotin‐associated enrichment of GABAA1, whereas in the post‐treatment assay, free HE showed limited ability to displace pre‐bound HE‐biotin‐GABAA1 complexes. These results support a specific and relatively stable interaction between HE‐biotin and GABAA1. To further assess whether HE‐biotin labeling is associated with surface‐accessible GABAA1, immunofluorescence staining was performed under non‐permeabilized conditions. HE‐biotin labeling was detected using streptavidin‐FITC, and GABAA1 was visualized by immunostaining. HE‐biotin generated a distinct surface‐associated signal that showed substantial overlap with GABAA1 immunoreactivity in SH‐SY5Y cells. The addition of excess unlabeled HE markedly diminished the streptavidin‐FITC signal, demonstrating that the HE‐biotin labeling was competitively blocked by the unlabeled compound (Figure 6B). Together with the competitive pull‐down results, these data support the specific association of HE‐biotin with surface‐accessible GABAA1‐related signals. The interaction between HE and GABAA1 was further examined using orthogonal target‐engagement assays. Microscale thermophoresis revealed a concentration‐dependent binding response between HE and GABAA1, with an apparent dissociation constant (*K_d_*) of 0.28 µm (Figure 6C). Consistently, a cellular thermal shift assay showed that HE increased the thermal stability of GABAA1 compared with the DMSO control, further supporting HE‐mediated engagement of GABAA1 (Figure 6D). We next investigated whether GABAA1 is functionally required for the inhibitory effect of HE in vivo. AAV‐mTH‐shRNA(*GABRA1*) (hereafter referred to as AAV‐mTH‐shGABAA1) was stereotactically delivered into the ventral tegmental area (VTA) to reduce GABAA1 expression preferentially in VTA dopaminergic neurons. Viral targeting and GABAA1 knockdown in the VTA were verified by immunofluorescence (Figure 6E). After VTA‐targeted GABAA1 knockdown, morphine still induced robust c‐Fos activation in the VTA, whereas HE no longer significantly reduced c‐Fos immunoreactivity relative to the Mor group (Figure 6F; ns). The overall group effect remained significant [*F*(4, 10) = 10.13, *p* = 0.0015, *η* ^2^ = 0.802; Table S2], reflecting the difference between the Con and Mor groups. Consistent with the c‐Fos results, morphine‐induced CPP remained detectable after VTA‐targeted GABAA1 knockdown. No overall group difference was detected at pretest [*F*(4, 25) = 1.28, *p* = 0.3044, *η* ^2^ = 0.170]. At test, the overall group effect was significant [*F*(4, 25) = 28.18, *p* < 0.0001, *η* ^2^ = 0.818], but none of the HE‐treated knockdown groups differed significantly from the Mor group (Figure 6G; ns; Table S2), indicating that the suppressive effect of HE was abolished after GABAA1 knockdown. These findings indicate that GABAA1 is required for HE‐mediated suppression of morphine‐induced neuronal activation and CPP. Importantly, the persistence of the CPP phenotype after GABAA1 knockdown suggests that GABAA1 signaling is not indispensable for CPP expression itself, but is necessary for the inhibitory effect of HE on morphine‐induced reward behavior. Collectively, these results identify GABAA1 as a functionally relevant HE‐associated target and demonstrate that GABAA1 in VTA dopaminergic neurons is required for HE‐mediated suppression of morphine‐induced neuronal activation and CPP.

### 2.6 Hederagenin Exerts an Anti‐Addiction Effect by GABAA1‐Dependent Inhibition of Ca^2+^–CaMK–cAMP–CREB Signaling

Chemical proteomics screening, followed by functional bioinformatics analysis of the filtered HE‐associated candidate protein set, identified GABAA1 as a prioritized candidate target of HE in morphine addiction. GO and KEGG enrichment analyses indicated that these candidate proteins were associated with cAMP signaling, calcium‐related signaling, and morphine addiction pathways, suggesting a potential link between HE target engagement and Ca^2^^+^–cAMP signaling regulation.

**FIGURE 7:**
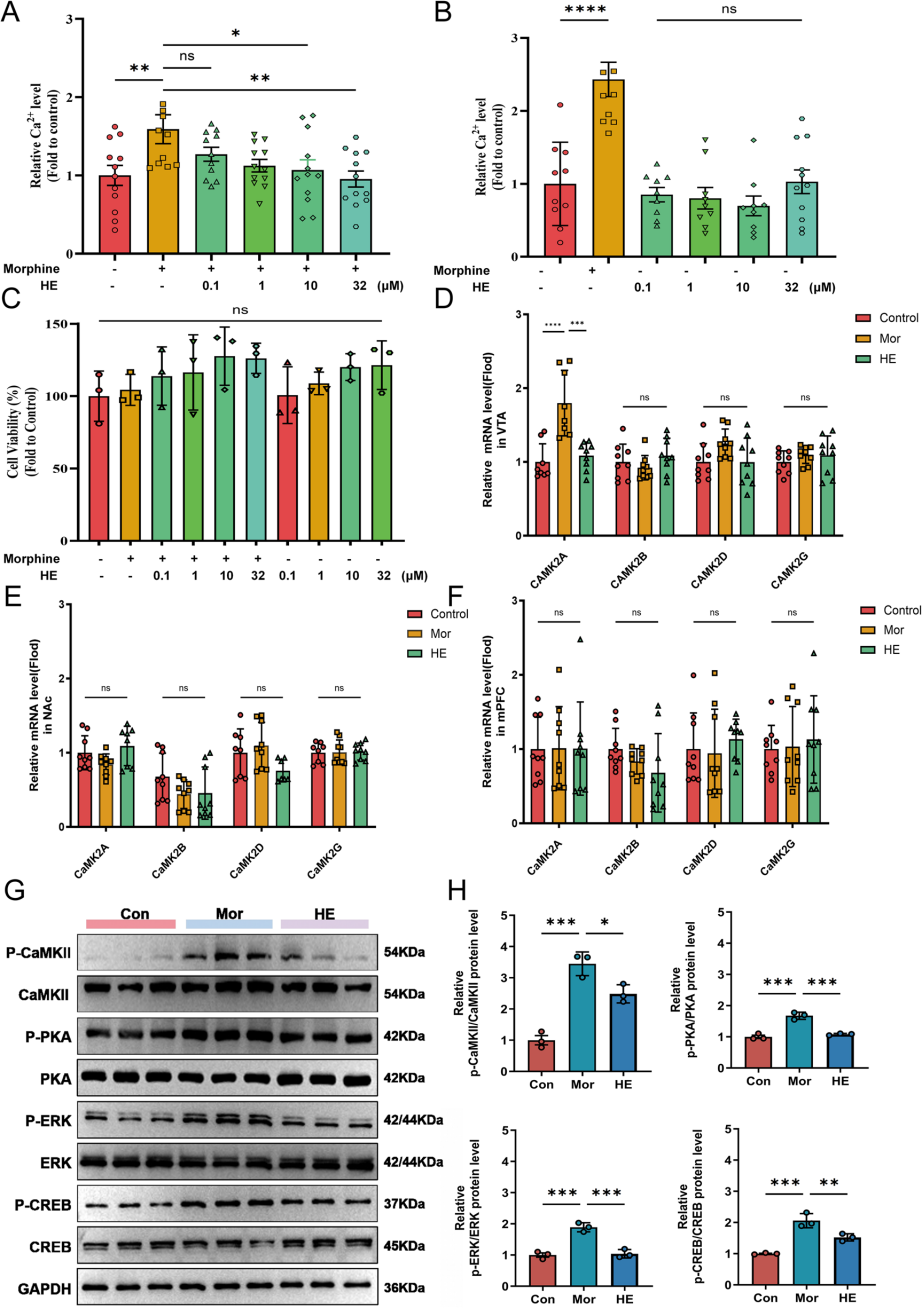
Hederagenin attenuates morphine‐induced Ca^2^^+^ elevation and suppresses CaMKII–cAMP–CREB pathway activation. (A) Intracellular Ca^2^^+^ levels in SH‐SY5Y cells after morphine exposure with or without HE treatment, measured using Fluo‐3 AM. HE attenuated morphine‐induced Ca^2^^+^ elevation across the tested concentrations. (B) Intracellular Ca^2^^+^ levels in SH‐SY5Y cells treated with HE alone in the absence of morphine, showing that HE itself did not markedly alter basal Ca^2^^+^ levels. (C) CCK‐8 assay showing the effect of HE on SH‐SY5Y cell viability after 48 h of treatment. Absorbance was measured at 450 nm and normalized to untreated control cells. (D–F) Quantitative PCR analysis of CaMKII isoform mRNA expression in the VTA, NAc, and mPFC regions. Morphine exposure significantly increased *Camk2a* mRNA expression in the VTA, which was reversed by HE treatment. (G) Representative Western blot images of p‐CaMKII, CaMKII, p‐PKA, PKA, p‐ERK, ERK, p‐CREB, CREB, and GAPDH in VTA tissue from the Con, Mor, and HE groups. (H) Quantification of relative phosphorylation levels in VTA tissue. Each phosphorylated protein signal was normalized to its corresponding total protein signal, followed by normalization to the mean value of the Con group. The Con group was set to 1 for relative comparison and does not represent complete phosphorylation. Data are presented as mean ± SEM. Panels A and H were analyzed using ordinary one‐way ANOVA followed by Dunnett's multiple‐comparisons test, with the Mor group as the reference. Panel B was analyzed using ordinary one‐way ANOVA with planned Dunnett comparisons, including prespecified comparisons between the Con group and HE‐alone groups. Panels C–F were analyzed using ordinary one‐way ANOVA followed by Tukey's multiple‐comparisons test. Each gene and phosphorylation ratio was analyzed separately. Sample sizes and the experimental unit are specified for the corresponding panels. Complete inferential statistics, including test statistics, degrees of freedom, exact *P‐*values, effect‐size estimates, and multiplicity‐adjusted comparisons, are provided in Table S2. ns, not significant; *p < 0.05; **p < 0.01; ***p < 0.001; ****p < 0.0001. Asterisks denote multiplicity‐adjusted *p‐*values for the comparisons indicated by the brackets. Each point represents one biological replicate.

Morphine markedly increased intracellular Ca^2^^+^ levels in SH‐SY5Y cells, whereas HE significantly attenuated this elevation at 1, 10, and 32 µm (*p < 0.05 to **p < 0.01; Figure 7A); the 0.1 µm comparison was not significant. The overall treatment effect was significant [*F*(5, 64) = 3.52, *p* = 0.0072, *η* ^2^ = 0.215; Table S2]. In the absence of morphine, the overall group effect was significant [*F*(5, 52) = 14.85, *p* < 0.0001, *η* ^2^ = 0.588], primarily reflecting the Mor–Con difference; however, the prespecified comparison between the Con group and HE alone at 32 µm was not significant (adjusted *p* > 0.9999; Figure 7B, Table S2), indicating that HE counteracted morphine‐induced Ca^2^^+^ dysregulation without directly disturbing basal calcium homeostasis. CCK‐8 analysis further showed that HE did not reduce cell viability/metabolic activity after 48 h of treatment (Figure 7C), suggesting that the Ca^2^^+^‐modulating effect of HE was not attributable to nonspecific cytotoxicity [*F*(9, 20) = 1.09, *p* = 0.4121, *η* ^2^ = 0.329; Figure 7C, Table S2]. To further examine whether HE regulates CaMK‐related signaling in vivo, we measured the mRNA expression of CaMKII isoforms in the VTA, NAc, and mPFC. In the VTA, *Camk2a* expression differed significantly among groups [*F*(2, 22) = 16.42, *p* < 0.0001, *η* ^2^ = 0.599], with morphine‐induced upregulation reduced by HE treatment (Figure 7D; ***p < 0.001). No significant overall group effects were detected for *Camk2b*, *Camk2d*, or *Camk2g* (all *p* > 0.05; Table S2). In the NAc, no significant overall group effects were detected for *Camk2a*, *Camk2b*, *Camk2d*, or *Camk2g* (all *p* > 0.05; Figure 7E, Table S2). In the mPFC, no significant group differences were observed for the tested CaMKII isoforms (all *p* > 0.05; Figure 7F, Table S2). These results suggest that the most prominent HE‐sensitive CaMKII isoform change occurred in *Camk2a*/CaMKIIα in the VTA.

Consistently, Western blot analysis of VTA tissue showed that morphine increased the relative phosphorylation levels of CaMKII, PKA, ERK, and CREB, whereas HE treatment reduced these phosphorylation changes (Figure 7G,H; *p < 0.05; **p < 0.01; ***p < 0.001). Significant overall group effects were detected for all four phosphorylation ratios [*F*(2, 6) = 38.05–54.67, *p* = 0.0001–0.0004, *η* ^2^ = 0.927–0.948; Table S2]. CPP scores were strongly positively correlated with the regional VTA p‐CREB/CREB ratio in the pooled matched dataset (two‐sided Pearson's *r* = 0.900, *p* < 0.0001, *n* = 15; Figure S4C). Treatment‐stratified analyses showed significant positive correlations in the Con group (*r *= 0.928, *p* = 0.0231) and Mor group (*r* = 0.995, *p* = 0.0005), whereas the Mor + HE group showed a directionally positive but non‐significant relationship (*r* = 0.850, *p* = 0.0679; *n* = 5 per group; Figure S4D). The CPP score × treatment group interaction was significant [*F*(2, 9) = 113.47, *p* < 0.0001, partial *η* ^2^ = 0.962], indicating that the regression slopes differed across treatment groups. Nevertheless, unlike the directional reversal observed for TH^+^/c‐Fos^+^ cell counts, the CPP score–p‐CREB/CREB relationships remained positive in direction in all three groups.

Collectively, these results support GABAA1 as a functionally relevant HE‐associated candidate target and indicate that HE attenuates morphine‐induced VTA Ca^2^^+^–CaMK–cAMP–CREB pathway activation. Together with the TH/c‐Fos co‐staining and AAV‐mTH‐G6S fiber photometry data, these findings suggest that the inhibitory effect of HE is associated with modulation of TH‐positive VTA dopaminergic neuronal activity during morphine‐induced CPP.

### 2.7 Preparation and Characterization of DSPE‐mPEG‐RVG29‐HE

**FIGURE 8:**
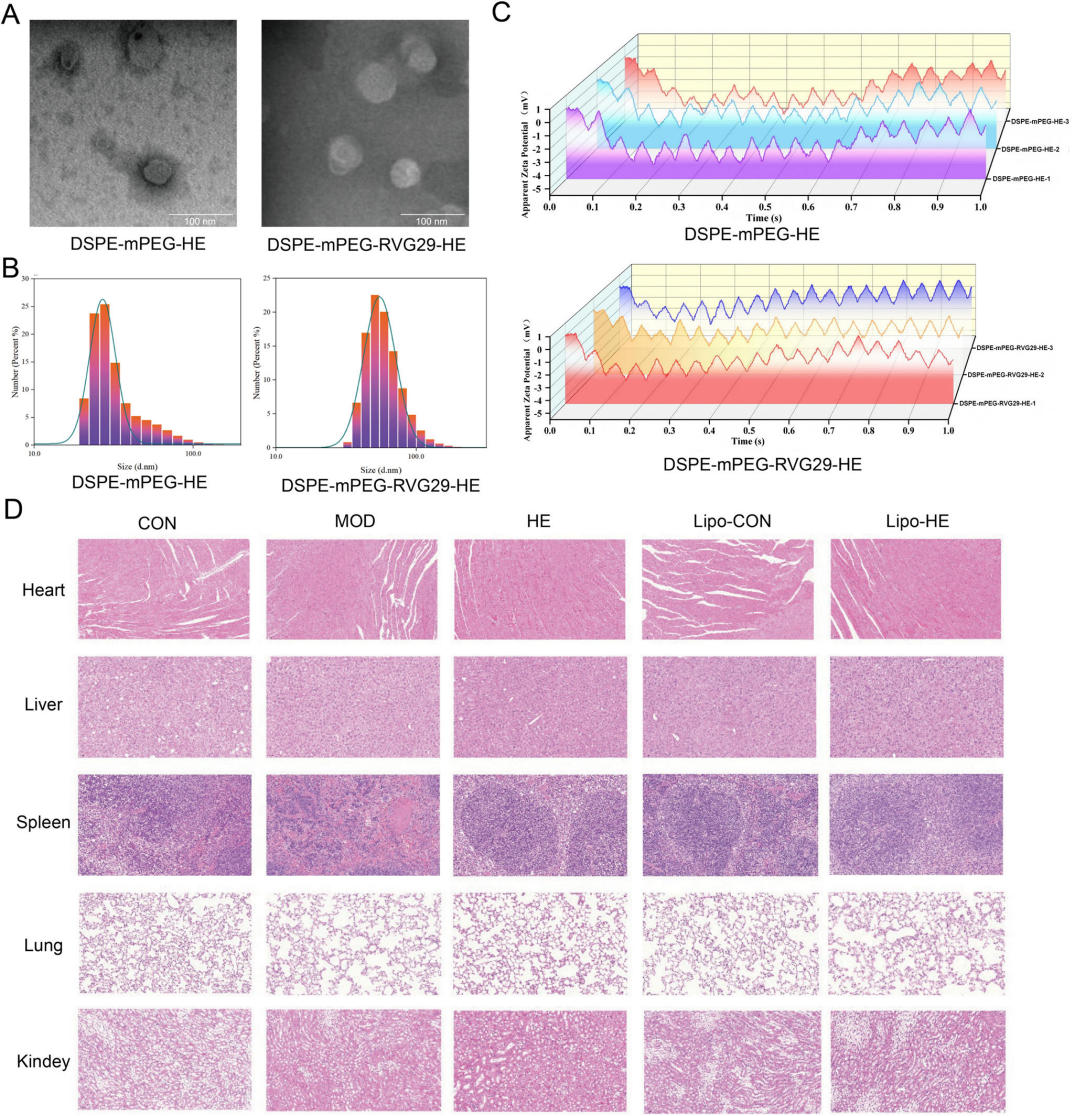
Preparation and physicochemical characterization of RVG29‐modified HE‐loaded liposomes. (A) Representative TEM images of DSPE‐mPEG‐HE and DSPE‐mPEG‐RVG29‐HE liposomes. (B) Particle‐size distributions. (C) Zeta potentials and short‐term size stability. (D) Representative H&E‐stained sections of major organs for safety assessment.

To improve the brain delivery of HE, RVG29‐modified HE‐loaded liposomes were prepared and characterized. Transmission electron microscopy (TEM) analysis showed that both non‐targeted liposomes (DSPE‐mPEG‐HE) and RVG29‐modified liposomes (DSPE‐mPEG‐RVG29‐HE) were uniformly dispersed nanoliposomes with smooth surfaces and no obvious aggregation (Figure 8A). The average particle size of DSPE‐mPEG‐HE was about 30 ± 3.2 nm, and the particle size increased to 73 ± 1.6 nm after RVG29 modification (Figure 8B). Dynamic light scattering (DLS) analysis showed that the zeta potential of DSPE‐mPEG‐HE was −4.4 ± 0.95 mV, whereas that of DSPE‐mPEG‐RVG29‐HE was −5.5 ± 3.66 mV, supporting the successful surface modification of RVG29 on the liposomes. In addition, the particle size of DSPE‐mPEG‐RVG29‐HE liposomes remained relatively stable within one week, indicating good short‐term colloidal stability of the formulation (Figure 8C).

To preliminarily assess the short‐term in vivo biosafety of the liposomal formulations, major organs, including the heart, liver, spleen, lungs, and kidneys, were collected after completion of the CPP behavioral test and subjected to histological examination. No obvious histopathological abnormalities were observed in the liposome‐treated groups compared with the control group (Figure 8D). The cell morphology and tissue structure remained intact and were comparable to those of the control group, suggesting that the liposome‐based treatment showed no obvious short‐term organ toxicity under the present experimental conditions.

### 2.8 Brain/VTA‐Associated Distribution of DSPE‐mPEG‐RVG29‐HE and Its Effect on Established Morphine CPP Expression

**FIGURE 9:**
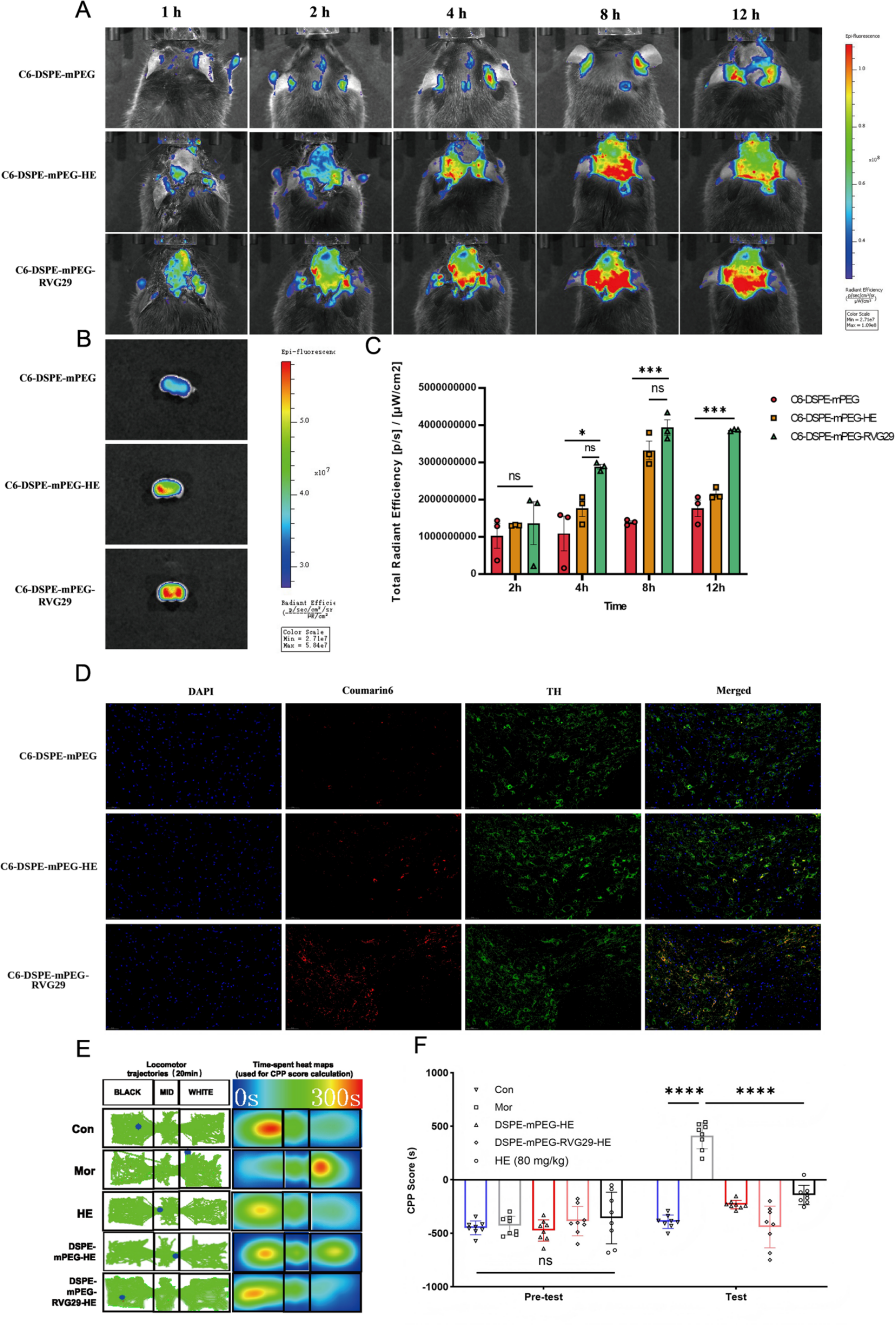
RVG29‐modified liposomes increase brain/VTA‐associated fluorescence distribution and enhance the acute suppression of established morphine CPP expression. (A) Serial in vivo fluorescence imaging of Coumarin 6‐labeled liposomes at the indicated time points. (B) Representative ex vivo fluorescence images of excised brains. (C) Quantification of fluorescence signals obtained by in vivo imaging. (D) Representative VTA sections showing DAPI, Coumarin 6, TH, and merged fluorescence. Coumarin 6/TH overlap indicates VTA‐associated distribution but does not demonstrate VTA‐specific delivery, intracellular HE release, or dopaminergic neuron‐specific uptake. (E) Representative locomotor trajectories during the 20‐min CPP test and corresponding time‐spent heat maps used for CPP score calculation. (F) CPP expression test evaluating the effects of free HE and HE‐loaded liposomal formulations on established morphine CPP. Morphine CPP was first established, and treatments were administered once before the post‐test. Liposomal formulations were administered via tail‐vein injection at 100 µL per mouse, containing approximately 0.27 mg HE per injection, corresponding to an HE‐equivalent dose of approximately 13.5 mg/kg for a 20 g mouse. This paradigm assessed acute modulation of established CPP expression rather than CPP acquisition. For panel C, data at each time point were analyzed separately using ordinary one‐way ANOVA followed by Tukey's multiple‐comparisons test. For panel F, pretest data and test data were analyzed using Welch's one‐way ANOVA followed by Dunnett's T3 multiple‐comparisons test, with the Mor group as the reference. Data are presented as mean ± SEM. Sample sizes and the experimental unit are specified for the corresponding panels. Complete inferential statistics, including test statistics, degrees of freedom, exact *P*‐values, effect‐size estimates, and multiplicity‐adjusted comparisons, are provided in Table S2. ns, not significant; *p < 0.05; **p < 0.01; ***p < 0.001; ****p < 0.0001. Asterisks denote multiplicity‐adjusted *p*‐values for the comparisons indicated by the brackets. Each data point represents one biological replicate.

The in vivo distribution of DSPE‐mPEG‐RVG29‐HE was evaluated using coumarin 6‐labeled liposomes combined with in vivo imaging and immunofluorescence analysis. As shown in Figure 9A, compared with the DSPE‐mPEG‐HE group, the DSPE‐mPEG‐RVG29‐HE group showed a stronger fluorescence signal in the brain region of mice. Quantification of fluorescence in excised whole‐brain tissue further showed that the fluorescence intensity was higher in the DSPE‐mPEG‐RVG29‐HE group than in the DSPE‐mPEG‐HE group (Figure 9B,C; ****p < 0.0001; ***p < 0.001; *p < 0.05), suggesting improved brain‐associated distribution after RVG29 modification. Separate one‐way ANOVAs showed no overall formulation effect at 2 h [*F*(2, 6) = 0.22, *p* = 0.8092, *η* ^2^ = 0.068], but significant effects at 4 h [*F*(2, 6) = 9.13, *p* = 0.0151, *η* ^2^ = 0.753], 8 h [*F*(2, 6) = 62.25, *p* < 0.0001, *η* ^2^ = 0.954], and 12 h [*F*(2, 6) = 58.34, *p* = 0.000117, *η* ^2^ = 0.951; Table S2]. To further examine the distribution of DSPE‐mPEG‐RVG29‐HE in the VTA region, TH was used to label dopaminergic neurons by immunofluorescence. In the VTA, the fluorescence signal of Coumarin 6‐loaded DSPE‐mPEG‐RVG29‐HE showed a punctate distribution and spatial overlap with TH‐positive regions, suggesting increased VTA‐associated distribution of the RVG29‐modified liposomes (Figure 9D). These fluorescence data indicate brain/VTA‐associated tissue distribution of labeled liposomes, but do not by themselves demonstrate intracellular HE release or functional uptake by dopaminergic neurons.

We next evaluated whether DSPE‐mPEG‐RVG29‐HE could enhance the acute effect of HE on established morphine CPP expression. Unlike the repeated acquisition‐phase administration shown in Figure 1, morphine CPP was first established through 5 days of conditioning, and the corresponding HE formulations were administered only once before the post‐test. During the baseline phase, no significant differences in baseline chamber preference were observed among groups. After 5 days of morphine conditioning, CPP was successfully established, as morphine‐treated mice showed a significantly increased preference for the drug‐paired compartment compared with the control group (Figure 9E,F). The corresponding HE formulations were then administered once before the post‐test to assess their effect on CPP expression. Compared with the model group, the DSPE‐mPEG‐HE group showed reduced CPP scores, suggesting that liposomal HE attenuated the expression of established morphine CPP (Figure 9F； ****p < 0.0001) [Welch's W(4, 16.48) = 65.63, p < 0.0001; Table S2]. Compared with DSPE‐mPEG‐HE, RVG29‐modified DSPE‐mPEG‐RVG29‐HE further reduced the CPP scores (Figure 9F). These results suggest that RVG29‐modified HE‐loaded liposomes enhanced the acute suppressive effect of HE on established morphine CPP expression under a single‐administration paradigm and produced a larger reduction in CPP scores than free HE at 80 mg/kg. To assess the animal‐level association between VTA fluorescence and behavioral outcome, CPP scores were matched with VTA Coumarin 6 mean fluorescence intensity (MFI) values from the same mice in the DSPE‐mPEG‐HE and DSPE‐mPEG‐RVG29‐HE groups. Across all eight matched animals, VTA Coumarin 6 MFI was significantly negatively correlated with CPP scores (Pearson *r* = −0.979, *p* < 0.0001; Figure S6A), indicating that higher VTA fluorescence was associated with greater CPP suppression. Formulation‐stratified analyses showed similar negative trends in the DSPE‐mPEG‐HE group (*r* = −0.929, *p* = 0.071; *n* = 4) and the DSPE‐mPEG‐RVG29‐HE group (*r* = −0.969, *p* = 0.031; *n* = 4; Figure S6B). Given the limited sample size, the stratified analyses were considered exploratory.

## 3 Discussion

Opioid use disorder remains a major public health challenge despite continued progress in overdose prevention, harm‐reduction strategies, and medication‐based treatment [16, 17]. Current evidence‐based medications, including methadone, buprenorphine, and naltrexone, are essential for opioid dependence management; however, pharmacological strategies that directly target opioid‐associated contextual reward learning, drug–context memory formation, and relapse‐related neural adaptations remain limited [17, 18, 19]. In this context, the present study investigated hederagenin (HE), a bioactive natural compound derived from *A. membranaceus*, as a candidate modulator of morphine‐associated contextual reward learning. Rather than positioning HE as a clinically validated anti‐addiction therapy, this study focuses on its effect on morphine‐CPP acquisition and the associated GABA_A_ receptor α1 subunit (GABAA1/*GABRA1*)‐related VTA Ca^2^^+^–CaMK–cAMP–CREB signaling mechanism.

HE was not selected arbitrarily but was prioritized through an initial functional screening strategy. Based on our previous identification of blood–brain barrier‐permeable active constituents from *A. membranaceus*, four candidate compounds—magnoflorine, cauloside C, HE, and oleanolic acid—were evaluated in parallel for their ability to modulate morphine‐induced intracellular cAMP accumulation. This screening was designed as a standardized comparative assay rather than a full concentration‐optimization study for each compound. Under the same concentration gradient, HE showed the clearest concentration‐dependent inhibitory trend on morphine‐induced cAMP reporter responses, providing a rational basis for advancing HE into behavioral and mechanistic studies.

The similar effects of HE and oleanolic acid in the initial cAMP screening should be interpreted carefully. Both compounds share a closely related oleanane‐type triterpenoid scaffold, and HE differs from oleanolic acid mainly by an additional hydroxyl group at C‐23 [20]. This structural difference may modestly influence polarity, solubility, and cellular accessibility under in vitro conditions, but the initial cAMP assay alone cannot establish that HE and oleanolic acid act through fundamentally distinct mechanisms. Therefore, the screening data should be viewed as a comparative functional screen rather than definitive evidence of mechanistic divergence between structurally related compounds. HE was prioritized because it showed a clearer concentration‐dependent inhibitory profile under standardized conditions.

The main behavioral finding of this study is that HE attenuated morphine‐induced CPP acquisition. This result should be interpreted within the appropriate conceptual boundary of the CPP model. CPP acquisition reflects the formation of a Pavlovian association between drug reward and a specific environmental context and provides a useful framework for studying opioid‐associated contextual reward learning [18, 19]. However, CPP acquisition does not directly measure psychological dependence‐like or relapse‐like behavior. To further examine whether HE affects already established morphine‐associated contextual preference, we added a CPP expression experiment in which HE was administered before the post‐test after CPP had been established. HE reduced the expression of established morphine CPP, suggesting that its behavioral effect was not strictly limited to acquisition. Nevertheless, because extinction/reinstatement and opioid self‐administration paradigms were not performed, these data should not be interpreted as direct evidence that HE prevents relapse‐like behavior or has therapeutic efficacy for opioid use disorder.

An important issue in CPP interpretation is whether reduced CPP scores reflect a selective effect on morphine‐associated contextual preference or nonspecific behavioral confounds. Several additional analyses support the former interpretation. HE alone did not produce major changes in sucrose preference, anxiety‐like behavior, spontaneous locomotor activity, exploratory behavior, or spatial learning/memory, as evaluated by the SPT, EPM, OFT, and MWM. In addition, raw compartment occupancy data from the baseline/pretest and test sessions showed that changes in CPP scores were not driven by increased time spent in the neutral compartment. Sex‐balanced behavioral validation further showed that HE attenuated morphine CPP acquisition in both male and female mice, and an additional matched conditioning‐time experiment reproduced its inhibitory effect. Together, these controls support that HE reduced morphine‐associated contextual preference rather than producing generalized behavioral suppression, anxiety‐related changes, cognitive impairment, sex‐restricted effects, or time‐of‐day confounding. The VTA is a central node in opioid‐associated reward processing and drug–context learning. Existing models emphasize that opioid reward involves mesolimbic dopamine signaling, local opioid receptor actions in the VTA, and interactions among dopaminergic, GABAergic, and glutamatergic neuronal populations [21, 22, 23]. In this study, morphine conditioning increased c‐Fos expression in addiction‐related brain regions, with a particularly HE‐sensitive change in the VTA. Because c‐Fos alone does not define cellular identity and may also be expressed under behavioral, exploratory, or stress‐related conditions, TH/c‐Fos double immunofluorescence was used to assess activity‐related changes in TH‐positive VTA dopaminergic neurons. Morphine increased the number of TH^+^/c‐Fos^+^ double‐positive cells in the VTA, whereas HE reduced this response.

In the matched TH/c‐Fos subset, CPP scores were positively associated with the number of VTA TH^+^/c‐Fos^+^ double‐positive cells when all animals were pooled across the Con, Mor, and Mor + HE groups. However, treatment‐stratified analyses showed that this pooled association was not uniform: the correlation was positive in the Mor group but negative in the Con and Mor + HE groups, with a significant CPP score × treatment group interaction. In morphine‐treated mice, the positive relationship is consistent with greater recruitment of VTA dopaminergic neurons in animals expressing stronger morphine‐conditioned preference. In contrast, when morphine‐conditioned preference was absent or attenuated, TH^+^/c‐Fos^+^ labeling may also reflect contextual salience, exploration, arousal, stress, or natural compartment preference. The negative relationship in the Mor + HE group should therefore not be interpreted as evidence that HE induced aversion, but may instead indicate that HE altered the relationship between CPP behavior and VTA dopaminergic neuronal activation. Given the limited subgroup sample sizes and the inability of TH/c‐Fos labeling to distinguish functionally or projection‐defined dopaminergic subpopulations, these findings should be interpreted as exploratory evidence of treatment‐dependent recruitment of VTA dopaminergic neurons.

In parallel, AAV‐mTH‐G6S fiber photometry enabled monitoring of population‐level calcium dynamics in VTA TH‐promoter‐targeted neurons during CPP behavior. Morphine enhanced compartment‐selective VTA calcium responses upon entry into the drug‐paired compartment, whereas HE attenuated this response. In the matched fiber‐photometry subset, higher CPP scores were associated with larger compartment‐selective VTA calcium responses, as reflected by the difference in post‐entry AUC between the drug‐paired and non‐drug‐paired compartments. This pooled animal‐level association suggests that stronger morphine‐associated contextual preference was accompanied by greater compartment‐selective VTA calcium activity. However, the analysis included only nine mice pooled across three experimental groups and may therefore reflect both between‐group separation and within‐group individual variability. Although positive slopes were observed in the Con, Mor, and Mor + HE groups, the sample size of three mice per group precluded reliable within‐group statistical inference. Moreover, correlation does not establish causality, and fiber photometry measures population‐level calcium dynamics rather than the activity of individual neurons. These results therefore provide supportive evidence of an association between CPP behavior and VTA calcium activity, rather than evidence that altered VTA calcium activity independently drives the behavioral phenotype.

A major mechanistic advance of this study is the identification of GABAA1/*GABRA1* as a prioritized HE‐associated candidate target. Chemical proteomics provided the initial target‐discovery framework. After excluding nonspecific proteins detected in the negative control, the HE‐biotin group was compared with the competitive control group to identify competitor‐sensitive candidate proteins. GABAA1 was highlighted from this filtered candidate set and was further supported by functional enrichment related to calcium and cAMP signaling, placing it within a candidate protein network connected to the signaling pathways altered in the cAMP and calcium assays.

The specificity of the HE–GABAA1 association was further supported by competition‐based validation. In competitive pull‐down assays, increasing concentrations of free unlabeled HE reduced HE‐biotin‐associated enrichment of GABAA1, especially under co‐treatment conditions, whereas post‐treatment competition showed limited displacement of pre‐bound HE‐biotin‐associated complexes. This suggests that HE‐biotin binding to GABAA1‐related protein complexes is competitor‐sensitive and relatively stable once formed. Consistently, non‐permeabilized HE‐biotin labeling showed surface‐associated HE‐biotin signals overlapping with GABAA1 immunoreactivity, and excess unlabeled HE markedly reduced the streptavidin‐FITC signal, supporting the specificity of HE‐biotin labeling at surface‐accessible GABAA1‐related sites rather than nonspecific probe accumulation. Orthogonal target‐engagement assays, including microscale thermophoresis and cellular thermal shift analysis, further supported HE–GABAA1 engagement. Given that α1‐containing GABA_A_ receptors possess extracellular α/γ subunit interfaces that are functionally relevant ligand‐binding regions for several allosteric modulators [24, 25], the extracellular *α*1(+)/*γ*2(−) interface was selected as the docking pocket and validated using a reference ligand. HE docking within this validated pocket provided structural plausibility for a HE–GABAA1‐associated interaction; however, docking remains a computational prediction and should not be interpreted as direct structural proof of binding.

The in vivo knockdown experiments provide functional relevance for GABAA1 in the behavioral and neural effects of HE. VTA‐targeted AAV‐mTH‐shGABAA1 reduced GABAA1 expression preferentially in TH‐positive VTA neurons. Under GABAA1‐knockdown conditions, HE no longer effectively suppressed morphine‐induced VTA c‐Fos activation or CPP scores, indicating that GABAA1 is required for the inhibitory effect of HE on morphine‐induced neuronal activation and CPP behavior. At the same time, morphine CPP remained detectable after GABAA1 knockdown, suggesting that GABAA1 is not indispensable for CPP expression itself. Instead, GABAA1 appears to function as a HE‐relevant modulatory node within the VTA rather than as the sole mechanism required for morphine‐associated contextual reward learning.

The proposed downstream mechanism links membrane‐associated GABAA1 modulation to Ca^2^^+^–CaMK–cAMP–CREB signaling. GABA_A_ receptor‐mediated inhibitory signaling is a key regulator of neuronal excitability and may influence calcium entry and downstream intracellular signaling through effects on membrane excitability [24]. In SH‐SY5Y cells, morphine increased intracellular Ca^2^^+^ levels, whereas HE attenuated this elevation. HE alone did not markedly alter basal Ca^2^^+^ levels or reduce CCK‐8‐derived cell viability/metabolic activity, suggesting that its Ca^2^^+^‐modulating effect was not simply due to nonspecific cytotoxicity or broad disruption of calcium homeostasis. In VTA tissue, morphine increased CaMKIIα expression and enhanced phosphorylation of CaMKII, PKA, and CREB, whereas HE reduced these changes. The involvement of cAMP/CREB and CaMKII‐related signaling is consistent with existing models of opioid‐induced molecular adaptation and reward‐associated learning [26, 27, 28, 29]. In contrast to the treatment‐dependent TH^+^/c‐Fos^+^ association, the relationship between CPP scores and the VTA p‐CREB/CREB ratio was directionally more consistent. CPP scores were strongly positively associated with the p‐CREB/CREB ratio in the pooled dataset, and positive relationships were observed in all three treatment groups, although the strength and statistical significance of the associations varied and the Mor + HE association did not reach statistical significance. This pattern is consistent with an association between regional VTA CREB signaling and morphine‐associated learning or activity‐dependent plasticity. Nevertheless, p‐CREB/CREB was measured in whole VTA tissue homogenates rather than specifically in TH‐positive neurons, and correlation does not establish causality or demonstrate that CREB selectively encodes reward‐memory consolidation. Taken together, these findings support a model in which HE acts through GABAA1‐related regulation of Ca^2^^+^‐dependent signaling to restrain downstream CaMK–cAMP–CREB activation associated with morphine CPP acquisition, whereas the c‐Fos findings indicate a more treatment‐dependent relationship between CPP behavior and recent activation of VTA dopaminergic neurons. These findings complement existing models of morphine‐associated reward learning rather than contradicting them. Established models emphasize that morphine reward involves mesolimbic dopaminergic activation, altered GABAergic inhibitory control, and CREB‐related intracellular plasticity. Our data support this framework by showing that morphine CPP acquisition was accompanied by enhanced VTA dopaminergic neuronal activation and increased Ca^2^^+^–CaMK–cAMP–CREB signaling. Importantly, the present study adds a potential upstream regulatory layer by identifying GABAA1 as a HE‐associated, membrane‐related modulatory node capable of linking inhibitory receptor engagement to Ca^2^^+^‐dependent intracellular signaling. Thus, the significance of HE lies not only in its behavioral effect on morphine CPP acquisition, but also in its ability to reveal a GABAA1‐related mechanism that may be pharmacologically targeted to modulate opioid‐associated contextual reward learning.

It is important to clarify the cellular interpretation of the signaling data. TH/c‐Fos staining and AAV‐mTH‐G6S photometry support the involvement of VTA TH‐positive dopaminergic neurons. However, qPCR and Western blot analyses were performed using VTA tissue and therefore do not provide cell‐type‐specific molecular measurements exclusively from dopaminergic neurons. Thus, the most rigorous interpretation is that HE reduces morphine‐induced Ca^2^^+^–CaMK–cAMP–CREB pathway activation in VTA tissue, while parallel TH/c‐Fos and fiber photometry data support the relevance of VTA dopaminergic neuronal activity. Future studies using fluorescence‐activated sorting of TH‐positive neurons, cell‐type‐specific ribosome profiling, single‐nucleus transcriptomics, or spatial proteomics would allow more precise assignment of these molecular changes to specific VTA neuronal subtypes.

The liposome experiments were designed to address a separate but related translational challenge: the limited BBB permeability of free HE. Many neuroactive natural products are limited by poor aqueous solubility, low bioavailability, rapid metabolism, or insufficient BBB penetration. Ligand‐modified nanocarriers have therefore been explored as strategies to improve CNS delivery. RVG/RVG29‐derived systems have been reported to facilitate brain delivery through nAChR‐related recognition, and RVG29‐functionalized lipid nanoparticles have been used in other brain‐delivery contexts [30, 31, 32]. In this study, RVG29‐modified HE‐loaded liposomes were developed to increase brain‐associated distribution and to evaluate whether RVG29 modification could enhance the acute effect of the HE‐loaded formulation in the CPP expression paradigm.

This liposome experiment differs from the repeated acquisition‐phase dosing paradigm. In the main acquisition experiment, HE was administered repeatedly during morphine‐paired conditioning sessions, allowing repeated pharmacological engagement during formation of the morphine–context association. In contrast, the liposome experiment was performed after CPP had already been established, and HE formulations were administered once before the post‐test. Therefore, the effect size of free HE in the liposome experiment should not be directly compared with the repeated acquisition‐phase effect as if the two experiments used the same behavioral design. The weaker effect of free HE under the single‐administration expression paradigm provides a rationale for evaluating formulation‐based delivery, although direct comparisons between oral and intravenous administration should be interpreted cautiously.

The delivery data provide preliminary support for improved brain/VTA‐associated distribution of the RVG29‐modified formulation. Compared with non‐targeted liposomes, RVG29‐modified liposomes showed stronger brain‐region fluorescence and greater spatial overlap with TH‐positive regions in the VTA. The time‐dependent fluorescence pattern should be interpreted cautiously. The increase in DSPE‐mPEG‐HE fluorescence up to 8 h followed by a decrease at 12 h likely reflects peak tissue accumulation followed by biological clearance, rather than continuous brain retention. In comparison, RVG29‐modified liposomes showed relatively sustained brain‐associated fluorescence during the observation window, suggesting that RVG29 modification may improve brain/VTA‐associated distribution of the formulation. The CPP expression experiment further suggested that RVG29‐modified HE‐loaded liposomes produced stronger suppression of established morphine CPP expression than non‐targeted liposomes under the single‐administration paradigm. Notably, the HE‐equivalent dose delivered by tail‐vein injection was lower than the free HE oral dose used in the gavage experiments, supporting the rationale that formulation‐based delivery may improve the effective utilization of HE. Therefore, these data provide preliminary evidence that RVG29 modification increases brain/VTA‐associated fluorescence distribution and may enhance the behavioral effect of the HE‐loaded formulation in the CPP expression paradigm. The matched animal‐level analysis further showed a significant negative association between VTA Coumarin 6 MFI and CPP scores across the DSPE‐mPEG‐HE and DSPE‐mPEG‐RVG29‐HE groups. Similar negative associations were observed within both formulation groups. Although the correlation in the DSPE‐mPEG‐RVG29‐HE group reached nominal statistical significance (P = 0.031), the formulation‐stratified analyses were considered exploratory because of the limited sample size (n = 4 per group). These findings provide preliminary evidence of an animal‐level relationship between regional fluorescence distribution and behavioral outcome. However, Coumarin 6 MFI reflects the tissue‐level distribution of the fluorescently labeled formulation and does not directly quantify intracellularly released or pharmacologically active HE. Moreover, this association does not establish exclusive VTA delivery, intracellular release, cell‐type‐specific uptake, or causality, particularly because the formulation was also distributed in other brain regions.

Several limitations should be acknowledged. First, the behavioral focus of this study was primarily CPP acquisition. Although an additional CPP expression experiment showed that HE reduced established morphine CPP expression, extinction/reinstatement and self‐administration paradigms were not performed. Therefore, the present findings cannot establish that HE prevents relapse‐like behavior or reduces compulsive opioid seeking. Future studies should evaluate HE in CPP extinction/reinstatement models, operant opioid self‐administration, progressive ratio reinforcement, and relapse‐like drug‐seeking paradigms. Second, although sex‐balanced behavioral validation showed that HE attenuated CPP acquisition in both male and female mice, the main mechanistic experiments were performed primarily in male mice. Larger sex‐balanced cohorts will be needed to evaluate possible sex‐dependent differences in HE efficacy, VTA signaling, and liposome delivery. Third, although multiple target‐engagement assays support GABAA1 as a prioritized HE‐associated candidate target, other HE‐associated proteins identified by chemical proteomics may also contribute to its effects. Natural products often act through polypharmacological mechanisms; therefore, GABAA1 should not be interpreted as the only possible target of HE. Future work should further validate additional candidate proteins and examine whether HE directly modulates GABAA1 receptor currents. Fourth, the proposed Ca^2^^+^–CaMK–cAMP–CREB mechanism was supported by cellular calcium assays, VTA tissue qPCR/Western blotting, and correlation analyses, but causality within this signaling cascade remains to be tested directly. Finally, the liposome delivery system requires further pharmacokinetic and mechanistic validation. The present fluorescence data support enhanced brain/VTA‐associated distribution of RVG29‐modified liposomes, but they do not directly quantify HE concentration in brain tissue or demonstrate intracellular release. Future studies should measure free and liposome‐associated HE in plasma and brain using LC–MS/MS, determine release kinetics under physiological conditions, and assess long‐term safety, immunogenicity, and off‐target tissue accumulation.

In summary, this study identifies HE as a candidate modulator of morphine‐associated contextual reward learning, with the strongest behavioral evidence centered on CPP acquisition. The data support a model in which HE engages membrane‐associated GABAA1‐related signaling, attenuates morphine‐induced VTA dopaminergic neuronal activation, and suppresses Ca^2^^+^–CaMK–cAMP–CREB pathway activation associated with morphine CPP. RVG29‐modified liposomes further provide an exploratory strategy to increase the brain‐associated distribution of the HE‐loaded formulation and enhance its effect under a single‐administration CPP expression paradigm. These findings provide a mechanistic foundation for future studies of HE and related delivery systems in opioid‐associated behavioral models, while additional relapse‐related paradigms, cell‐type‐specific mechanistic studies, and pharmacokinetic validation are required before broader translational conclusions can be drawn.

## 4 Experimental Section

### 4.1 Chemicals and Reagents

Hederagenin (HE, purity ≥98%) was purchased from Taoshu (China) and dissolved in dimethyl sulfoxide (DMSO) to prepare stock solutions. For all in vitro experiments, HE was freshly diluted in culture medium to the indicated concentrations, with the final DMSO concentration maintained below 0.1%. Morphine hydrochloride was obtained from the National Institute for the Control of Pharmaceutical and Biological Products (China). Fluo‐3 AM was purchased from Yeasen (China). Streptavidin‐conjugated magnetic beads were obtained from MedChemExpress (MCE). Information on primary and secondary antibodies is provided in the Supporting Information. All other reagents were of analytical grade or higher.

### 4.2 SH‐SY5Y Cell Culture

Human neuroblastoma SH‐SY5Y cells were maintained in Dulbecco's modified Eagle's medium (DMEM; KeyGEN BioTECH) supplemented with 10% fetal bovine serum (FBS; Bio‐Channel), 100 µg/mL penicillin, and 100 µg/mL streptomycin. Cells were cultured at 37°C in a humidified incubator containing 5% CO_2_ and passaged at approximately 70%–80% confluence. To minimize phenotypic variation, cells between passages 5 and 20 were used.

For fluorescence‐based assays, cells were seeded at a density of 1 × 10^4^ cells per well in black‐walled, clear‐bottom 96‐well plates and allowed to adhere overnight prior to drug treatment. Vehicle control cells received 0.1% DMSO.

### 4.3 Measurement of Intracellular cAMP Dynamics

Intracellular cAMP dynamics were measured using the pGloSensor‐22F cAMP reporter system (Promega, USA). SH‐SY5Y cells were transiently transfected with the pGloSensor‐22F plasmid using Lipofectamine 3000. Twenty‐four hours after transfection, cells were replated into white 96‐well plates at a density of 4–5 × 10^4^ cells per well and allowed to recover for 12 h. Cells were then incubated with morphine (5 µm) in the presence or absence of the indicated compounds for 24 h.

For the initial comparative screening assay, four candidate compounds from *A. membranaceus*, including magnoflorine, cauloside C, hederagenin, and oleanolic acid, were tested using the same twofold concentration gradient of 8, 16, and 32 µm to ensure direct comparability across compounds under standardized conditions. The candidate compounds and concentration range were selected based on our previous identification of blood–brain barrier‐permeable Astragalus‐derived constituents, the reported pharmacological relevance of these compounds, and commonly used SH‐SY5Y cellular assay dosing ranges for these or structurally related compounds [33, 34, 35]. This range was used as a practical low‐to‐moderate micromolar screening window for evaluating relative efficacy and potential concentration‐dependent effects while minimizing nonspecific or cytotoxic interference at higher concentrations.

Baseline luminescence was recorded at 1 min intervals for 10 cycles, followed by forskolin stimulation and continuous luminescence recording at 1 min intervals for an additional 35 cycles. cAMP reporter responses were evaluated from the real‐time luminescence traces and quantified by area under the curve (*AUC*) analysis.

### 4.4 Animals and Ethical Approval

C57BL/6J mice aged 8–10 weeks and weighing 20–25 g were used in this study. Unless otherwise stated, male mice were used for the in vivo and behavioral experiments. For the additional CPP behavioral validation experiment designed to evaluate the potential influence of sex, both male and female C57BL/6J mice were included; female mice were also 8–10 weeks of age and weighed 20–25 g, and sex was balanced across experimental groups. Animals were housed under standard laboratory conditions with a 12 h light/dark cycle, controlled temperature and humidity, and free access to food and water. All mice were allowed to acclimate to the animal facility for at least 7 days before the experiments. All animal procedures were performed in accordance with the guidelines for the care and use of laboratory animals and were approved by the Institutional Animal Care and Use Committee of Nanjing University of Chinese Medicine under approval number [202501A050].

### 4.5 Conditioned Place Preference (CPP)

CPP was performed using a biased three‐compartment apparatus consisting of two conditioning chambers (15 cm × 18 cm × 20 cm) connected by a neutral compartment (15 cm × 8 cm × 20 cm). The two conditioning chambers differed in both visual and tactile cues: one chamber had black walls with a striped floor, whereas the other had white walls with a metal mesh floor. Sliding doors were used to restrict access during conditioning sessions.

The CPP procedure consisted of three phases: pretest, conditioning, and test. During the pretest phase, mice were allowed free access to all compartments for 20 min, and the time spent in each chamber was recorded to determine baseline preference. In this biased design, the initially non‐preferred chamber was assigned as the drug‐paired chamber. Jitai tablets were used as a positive‐control treatment in the morphine‐induced CPP experiment. Jitai tablets are a traditional Chinese medicine formulation used clinically for opioid detoxification. In this study, Jitai tablets were suspended in vehicle and administered by oral gavage at 0.087 g/kg during the conditioning phase [36].

For the morphine‐induced CPP experiment, mice were randomly assigned to the control, morphine model, low‐dose HE, medium‐dose HE, high‐dose HE, or Jitai tablet groups. Morphine was administered intraperitoneally at 10 mg/kg as the conditioning stimulus. HE was administered by oral gavage at 20, 40, or 80 mg/kg, and Jitai tablets were administered by oral gavage at 0.087 g/kg as a positive control. On morphine‐paired conditioning sessions, HE or Jitai tablets were given 30 min before morphine injection, after which mice were confined to the drug‐paired chamber for 30 min. On alternate conditioning sessions, mice received the corresponding vehicle treatment followed by saline conditioning in the opposite chamber according to a standard alternating CPP schedule. Each group received the same number of morphine‐paired and saline‐paired conditioning sessions.

The conditioning phase was performed for 5 consecutive days. To minimize potential confounding by unequal time‐of‐day exposure, conditioning sessions were conducted according to a fixed schedule, and an additional validation experiment was performed in which the low‐dose and high‐dose HE groups were conditioned at the same time of day under otherwise matched conditions. This repeated experiment yielded the same overall behavioral conclusion.

On the test day, mice were allowed free access to all compartments for 20 min in the absence of drug treatment. Behavioral activity was recorded using the VisuTrack animal behavior analysis system (XinRuan Co., Ltd., Shanghai, China). CPP scores were calculated as the time spent in the drug‐paired chamber minus the time spent in the non‐drug‐paired compartment. Pretest and test data were analyzed separately.

To assess whether the behavioral effect of HE was restricted to male mice, an additional CPP acquisition validation experiment was performed in both male and female C57BL/6J mice. Mice of each sex were assigned to the Con, Mor, HE‐LD, and HE‐HD groups. The CPP protocol was conducted as described above. HE was administered by oral gavage 30 min before morphine injection during morphine‐paired conditioning sessions. HE‐LD and HE‐HD indicate HE at 20 and 80 mg/kg, respectively. CPP scores were calculated as the time spent in the drug‐paired compartment minus the time spent in the non‐drug‐paired compartment during the same test session.

### 4.6 Behavioral Assessment of Potential Confounding Effects of HE

To determine whether HE alone induced behavioral alterations that might confound the interpretation of morphine CPP, an independent cohort of mice was subjected to a series of behavioral tests following HE treatment alone. Mice were randomly assigned to four groups: control (Con), low‐dose HE (LD, 20 mg/kg), medium‐dose HE (MD, 40 mg/kg), and high‐dose HE (HD, 80 mg/kg), with *n* = 5 mice per group. HE was administered by gavage at the same doses used in the CPP experiments, whereas control mice received an equal volume of vehicle. All treatments and behavioral tests were performed within the same time window each day.

### 4.7 Sucrose Preference Test

The SPT was performed to assess natural reward preference. The procedure included adaptation, water deprivation, and testing phases. During testing, mice were provided with one bottle containing sucrose solution and one bottle containing water. HE or vehicle was administered at the beginning of the test. Bottle positions were switched midway through the test to avoid side preference. Total fluid intake and sucrose preference were recorded at 12 and 24 h [37].

### 4.8 Elevated Plus Maze (EPM)

For the EPM, mice were placed in the center of the maze facing an open arm 30 min after vehicle or HE administration and allowed to explore for 5 min. The number of entries into the open arms, closed arms, and center, as well as the time spent in each zone, were recorded using the VisuTrack animal behavior analysis system (XinRuan Co. Ltd., Shanghai, China) [38].

### 4.9 Morris Water Maze (MWM)

For the MWM, mice received vehicle or HE 30 min before each daily training session. Mice were trained for four consecutive days and tested on the fifth day. Latency to find the platform, distance to the platform, and swimming speed were recorded using the VisuTrack animal behavior analysis system (XinRuan Co. Ltd., Shanghai, China). Data from each training day were analyzed separately to compare group differences.

### 4.10 Open‐Field Test

For the OFT, mice were placed individually into the open field 30 min after vehicle or HE administration and allowed to explore freely for 10 min. Total distance traveled, activity time percentage, and number of center entries were recorded using the VisuTrack animal behavior analysis system (XinRuan Co. Ltd., Shanghai, China) [39].

### 4.11 Biotin‐Labeled HE Probe Synthesis

A biotinylated hederagenin probe (HE‐biotin, Bio‐HE) was synthesized by introducing a linker at the C‐28 position of HE followed by covalent conjugation to biotin. The chemical structure and purity of the probe were confirmed by NMR and HRMS before use [40].

### 4.12 Chemical Proteomics and LC–MS/MS Analysis

SH‐SY5Y cell lysates were prepared in ice‐cold RIPA buffer containing protease inhibitors. Equal amounts of total protein (1–2 mg) were assigned to three groups: negative control, biotin group, and competitive control. The biotin group was incubated with HE‐biotin at 4°C for 2 h, while the competitive control group was incubated with HE‐biotin in the presence of excess unlabeled HE. The negative control group was processed without HE‐biotin. Probe‐associated proteins were enriched using streptavidin magnetic beads, washed extensively, eluted, and separated by SDS–PAGE. Gel bands were excised, subjected to in‐gel tryptic digestion, and analyzed by LC–MS/MS, with database searches performed with an *FDR* < 1%. Proteins detected in the negative control were first excluded as nonspecific background. Differential proteomic analysis was then primarily based on the comparison between the biotin group and the competitive control group, and GO/KEGG enrichment analyses were performed on the filtered candidate protein set.

### 4.13 Competitive Pull‐Down and Immunoblotting

To assess binding specificity, cell lysates were pre‐incubated with excess unlabeled HE prior to incubation with HE‐biotin. Pull‐down eluates were separated by SDS–PAGE and analyzed by immunoblotting to detect candidate target proteins.

### 4.14 Molecular Docking

Molecular docking was performed using AutoDock4 with the Lamarckian genetic algorithm (LGA). The receptor structure used for docking was the experimentally resolved cryo‐EM structure of the human full‐length α1β3γ2L GABA_A_ receptor (PDB ID: 6HUG), which was prepared and saved as 6HUG_pymol.pdbqt. During receptor preparation, water molecules, unrelated ligands, and irrelevant ions were removed. Polar hydrogen atoms and Kollman charges were added using AutoDock Tools. The 3D structure of hederagenin (HE) was obtained from PubChem and prepared as the ligand file HE.pdbqt with Gasteiger charges and defined rotatable bonds using AutoDock Tools. The docking pocket was not defined solely by an unconstrained cavity search. Instead, based on published structural information for the 6HUG receptor, the selected pocket was defined in the benzodiazepine‐binding region at the extracellular *α*1(+)/*γ*2(−) interface (Figure S3). The docking grid box was centered at *x* = 135.525, *y* = 139.346, *z* = 137.153, with dimensions of 28.67 × 28.67 × 28.67 Å. To provide a basic validation of the docking protocol, diazepam was docked into the same selected pocket using the same receptor structure and docking parameters as those subsequently applied to HE. For each ligand, 100 independent docking runs were performed. The output docking file for HE was saved as HE_out.pdbqt, and the top‐ranked pose was selected for interaction analysis. The reported docking value of −10 kcal/mol represents the best‐scoring predicted docking pose generated under the applied docking conditions, rather than an experimentally measured binding affinity. Ligand–receptor interactions were visualized using PyMOL, and residues identified in the docking model were interpreted as predicted interacting residues.

### 4.15 Non‐Permeabilized Immunofluorescence Staining and HE‐Biotin Competition Assay

To assess the surface‐associated labeling pattern of HE‐biotin and its association with GABAA1, SH‐SY5Y cells were seeded on confocal dishes or glass coverslips and allowed to adhere overnight. Cells were incubated with HE‐biotin under the indicated conditions. For the competition group, cells were co‐incubated with excess free unlabeled HE to evaluate whether the HE‐biotin labeling signal could be competitively reduced by the parent compound. After treatment, cells were gently washed with PBS and fixed with 4% paraformaldehyde. Importantly, the staining was performed under non‐permeabilized conditions, and no detergent permeabilization step was included. This procedure was used to preferentially assess surface‐associated HE‐biotin labeling. Cells were blocked with BSA‐containing blocking buffer and incubated with an anti‐GABAA1 primary antibody, followed by the corresponding fluorescent secondary antibody. HE‐biotin labeling was detected using streptavidin‐FITC. Nuclei were counterstained with DAPI. Fluorescence images were acquired using the same microscope settings across groups. DAPI was used only as a nuclear counterstain, while streptavidin‐FITC was used to detect HE‐biotin labeling. The decrease in streptavidin‐FITC signal after excess unlabeled HE competition was used to support the specificity of HE‐biotin labeling. Because no permeabilization was performed, the observed HE‐biotin signal was interpreted as surface‐associated labeling rather than generalized intracellular staining. DAPI is a DNA‐binding nuclear counterstain and should not be interpreted as a plasma membrane marker.

### 4.16 Microscale Thermophoresis (MST)

HEK293T cells were transiently transfected with a 3×FLAG‐GABAA1‐EGFP expression plasmid. Membrane proteins were extracted and incubated with serial dilutions of HE. Thermophoretic measurements were performed using a Monolith NT.115 Pico instrument (NanoTemper Technologies), and dissociation constants (*K_d_*) were calculated using the accompanying analysis software.

### 4.17 Stereotaxic AAV Injection Into the VTA

For VTA‐targeted viral manipulation, mice were anesthetized with 2% isoflurane and placed in a stereotaxic apparatus (RWD, China). The scalp was incised along the midline to expose the skull, and erythromycin ophthalmic ointment was applied to protect the eyes. Viral vectors were stereotactically injected into the ventral tegmental area (VTA) using a glass micropipette with a diameter of approximately 0.2 mm. For in vivo fiber photometry recording, AAV‐mTH‐G6S was injected into the VTA to express GCaMP6s in TH‐promoter‐targeted dopaminergic neurons. In this construct, the mTH promoter was used to drive GCaMP6s expression preferentially in TH‐positive neurons, and G6S refers to GCaMP6s, a genetically encoded calcium indicator. For in vivo functional interrogation of GABAA1, AAV‐mTH‐shGABAA1 was injected into the VTA to reduce GABAA1 expression preferentially in TH‐positive dopaminergic neurons. Before injection, the microinjection pump was set to withdrawal mode to load 500 nL of viral solution at a withdrawal speed of 500 nL/min. After loading, the micropipette was positioned above the target brain region and slowly lowered to the VTA injection depth of −4.25 mm. Viral solution was then infused into the VTA at a total volume of 500 nL and an infusion rate of 50 nL/min. After infusion, the micropipette was left in place for 10 min to allow viral diffusion and minimize reflux, and was then slowly withdrawn.

For fiber photometry experiments, an optical fiber was implanted above the VTA after viral injection to record calcium signals during CPP behavior. For GABAA1 knockdown experiments, mice were allowed at least 3 weeks for viral expression before subsequent behavioral and histological analyses. Viral expression and injection sites in the VTA were verified histologically after the experiments.

### 4.18 Fiber Photometry Data Analysis

Fiber photometry was conducted in a dedicated subset of the overall CPP cohort, comprising three mice each from the Con, Mor, and Mor + HE groups (*n* = 9 in total). For each mouse, calcium activity was quantified as the *AUC* of the event‐aligned Δ*F*/*F* signal during the 0–10 s interval following entry into the drug‐paired or non‐drug‐paired compartment. Repeated compartment‐entry events from the same mouse were summarized at the animal level and were not treated as independent biological replicates. The compartment‐selective calcium‐response index was calculated as Δ*AUC *= *AUC*(drug‐paired) − *AUC*(non‐drug‐paired). CPP scores and Δ*AUC* values obtained from the same mice were used for the animal‐level correlation analysis.

### 4.19 Quantitative Real‐Time PCR and Western Blot

Total RNA was extracted from mouse VTA tissues using TRIzol reagent and reverse‐transcribed into cDNA. Quantitative real‐time PCR was performed using a SYBR Green system, and relative expression levels were calculated using the 2^−^ *^ΔΔCt^* method with *Gapdh* as the internal reference gene. The specific primers utilized are detailed in Table S1.

For Western blot analysis, protein samples were separated by SDS–PAGE and transferred to PVDF membranes. Membranes were blocked with 5% bovine serum albumin (BSA) and incubated with primary antibodies at 4°C overnight, followed by incubation with HRP‐conjugated secondary antibodies. Bands were visualized using enhanced chemiluminescence and quantified by densitometry.

### 4.20 Measurement of Intracellular Ca^2^^+^ Levels

Intracellular Ca^2^^+^ levels were measured using the Fluo‐3 AM fluorescence assay. SH‐SY5Y cells were treated according to the indicated experimental design. After treatment, the culture medium was removed, and cells were washed three times with HBSS buffer. Fluo‐3 AM stock solution was diluted with HBSS to a final concentration of 4 µm, and 100 µL Fluo‐3 AM working solution was added to each well. Cells were incubated at 37°C for 45 min in the dark. After dye loading, the Fluo‐3 AM working solution was removed, and cells were washed three times with HBSS. Each well was then covered with 100 µL HBSS and incubated at 37°C for 20 min.

Baseline fluorescence intensity (*F*) was measured using a microplate reader, and each group was measured in triplicate. To determine maximal fluorescence (*F*_max_), CaCl_2_ was added to each well at a final concentration of 1 mm. To determine minimal fluorescence (*F*_min_), the solution was removed, and EGTA was added at a final concentration of 5 mm. Intracellular Ca^2^^+^ concentration was calculated using the following formula:

$${\left[Ca^{2+}\right]}_{i}=K_{d}\times \frac{F-F_{\min}}{F_{\max}-F}$$

where *K_d_* is the dissociation constant of Fluo‐3 AM and was set to 400 nm.

### 4.21 Cell Viability Assay

Cell viability was assessed using the Cell Counting Kit‐8 (CCK‐8) assay. SH‐SY5Y cells were seeded in black clear‐bottom 96‐well plates at a density of 5 × 10^4^ cells/mL, with 100 µL cell suspension added to each well. Cells were treated according to the indicated drug‐treatment protocol for 48 h, and each group contained six replicate wells. After treatment, 10 µL CCK‐8 solution was added to each well and gently mixed to avoid bubble formation. The plates were incubated at 37°C in 5% CO_2_ for 45 min, and absorbance was measured at 450 nm using a microplate reader.

Blank wells containing culture medium and CCK‐8 reagent without cells were used for background correction. Relative cell viability was calculated as follows:

$$\mathrm{Cell}\;\mathrm{viability}\;\left(\%\right)=\frac{A_{\mathrm{treated}}\;-\;A_{\mathrm{blank}}}{A_{\mathrm{control}}\;-\;A_{\mathrm{blank}}}\;\times \;100$$

where *A*_treated_ represents the absorbance of drug‐treated wells, *A*_control_ represents the absorbance of untreated control wells, and *A*_blank_represents the absorbance of blank wells.

### 4.22 Preparation and Characterization of DSPE‐mPEG‐RVG29‐HE

HE‐loaded liposomes were prepared using the thin‐film hydration extrusion method as previously described with minor modifications. Briefly, lipid components for non‐targeted liposomes and RVG29‐modified targeted liposomes were dissolved together with hederagenin (HE) in an organic solvent to form a homogeneous lipid solution. The organic solvent was removed under reduced pressure to generate a thin lipid film, which was further dried to remove residual solvent. The dried lipid film was hydrated with aqueous buffer, followed by sonication and extrusion to obtain uniformly dispersed liposomes. For non‐targeted HE‐loaded liposomes, the formulation was prepared without RVG29 modification and was designated as DSPE‐mPEG‐HE. For targeted liposomes, RVG29‐modified lipid was incorporated into the formulation to generate DSPE‐mPEG‐RVG29‐HE. RVG29 was used as a targeting ligand based on its reported ability to interact with nicotinic acetylcholine receptors and facilitate BBB transport and neuronal delivery. Unencapsulated HE was removed before subsequent characterization and biological experiments. The morphology of liposomes was observed by transmission electron microscopy (TEM). Briefly, liposome samples were diluted appropriately, dropped onto copper grids, negatively stained, dried at room temperature, and imaged using TEM. Particle size distribution and zeta potential were measured by DLS. To evaluate formulation stability, DSPE‐mPEG‐RVG29‐HE liposomes were stored under the indicated conditions, and particle size changes were monitored over one week [41, 42].

### 4.23 Liposome Administration

For the CPP expression experiment evaluating liposomal HE delivery, mice first underwent the standard morphine CPP conditioning procedure to establish CPP. After CPP was established, free HE, non‐targeted HE‐loaded liposomes, or RVG29‐modified HE‐loaded liposomes were administered once before the post‐test. According to the formulation calculation, each mouse received 100 µL of liposome formulation by tail‐vein injection, containing approximately 0.27 mg HE per mouse, corresponding to an HE‐equivalent concentration of approximately 2.7 mg/mL and an HE‐equivalent dose of approximately 13.5 mg/kg for a 20 g mouse. Free HE was administered at 80 mg/kg. Liposomal formulations were administered intravenously via the tail vein 30 min before the post‐test. Mice were then allowed free access to the CPP apparatus. For each test session, the CPP scores were calculated as the time spent in the drug‐paired compartment minus the time spent in the non‐drug‐paired compartment. For the animal‐level fluorescence–behavior correlation analysis, the post‐test CPP score of each imaged mouse was matched with its corresponding VTA Coumarin 6 MFI value. This experiment was designed to evaluate the acute effect of HE formulations on the expression of established morphine CPP rather than CPP acquisition.

### 4.24 Immunofluorescence Image Quantification

Immunofluorescence images were acquired using identical microscope settings within each staining experiment. Anatomically matched regions of interest were selected across groups for quantification. For c‐Fos analysis, c‐Fos‐positive nuclei in the VTA, NAc, and Hip were quantified using a semi‐automated approach in ImageJ/Fiji. After background subtraction, the same thresholding criteria were applied to all images within each staining set. c‐Fos‐positive nuclei were initially detected using the particle analysis function in ImageJ/Fiji based on predefined size and circularity criteria, followed by manual verification to exclude nonspecific signals, artifacts, or overlapping structures. The analysis was performed by an investigator blinded to the experimental groups. For TH/c‐Fos double immunofluorescence analysis in the VTA, TH‐positive dopaminergic neurons and c‐Fos‐positive nuclei were first identified from the corresponding single‐channel images using the same thresholding criteria across groups. TH^+^/c‐Fos^+^ double‐positive cells were then determined based on signal overlap in the merged images and manually verified in ImageJ/Fiji. The number of TH^+^/c‐Fos^+^ cells was quantified from matched VTA fields. For each mouse, values from matched sections or fields were averaged before statistical analysis, and each biological replicate represented one mouse rather than one image field. For the liposome‐distribution analysis, Coumarin 6 MFI was quantified within anatomically matched VTA regions of interest using ImageJ/Fiji. Images were acquired using identical microscope settings across the two formulation groups. For each mouse, MFI values obtained from the analyzed VTA sections or fields were averaged to generate one animal‐level MFI value, which was then matched to the CPP score obtained from the same individual mouse. Only mice in the DSPE‐mPEG‐HE and DSPE‐mPEG‐RVG29‐HE groups with complete matched behavioral and fluorescence data were included in the correlation analysis. Each data point therefore represented one biologically independent mouse.

### 4.25 Statistical Analysis

Data are presented as mean ± SEM. Statistical analyses were performed using GraphPad Prism 10.6.0 (GraphPad Software, USA). Homogeneity of variance was assessed using the Brown–Forsythe test before parametric analyses. Matched two‐group data were analyzed using a paired two‐tailed Student's *t*‐test. Independent two‐group data were analyzed using an unpaired two‐tailed Student's *t*‐test when equal variances were assumed or Welch's *t*‐test when equal variances were not assumed. For multiple‐group comparisons, ordinary one‐way ANOVA followed by Tukey's multiple‐comparisons test was used for all pairwise comparisons, whereas Dunnett's multiple‐comparisons test was used when treatment groups were compared with a prespecified reference group. When equal variances were not assumed, Welch's one‐way ANOVA followed by Dunnett's T3 multiple‐comparisons test was used. Pretest and test data and data obtained at different time points were analyzed separately where indicated. Matched animal‐level associations were assessed using two‐sided Pearson correlation analyses, and treatment‐dependent associations were examined using linear‐model interaction analyses. All tests were two‐sided, and *p* < 0.05 was considered statistically significant. Exact *p*‐values are reported except when *p* < 0.0001. Effect‐size estimates included Cohen's *d*_z_ for paired *t*‐tests, *η* ^2^ for one‐way analyses, partial *η* ^2^ for interaction terms, and Pearson's *r* for correlation analyses. Complete inferential statistics, including test statistics, degrees of freedom, exact *P*‐values, effect‐size estimates, and multiplicity‐adjusted comparisons, are provided in Table S2.

Animal‐level correlation analyses were performed using behavioral and molecular or fiber‐photometry measurements individually matched within the same mice. Two‐sided Pearson correlation coefficients were calculated to assess the associations of CPP scores with VTA TH^+^/c‐Fos^+^ double‐positive cell counts, the VTA p‐CREB/CREB ratio, and the compartment‐selective calcium‐response index (Δ*AUC*). For the molecular analyses, correlations were calculated for the pooled dataset and separately within the Con, Mor, and Mor + HE groups. Exploratory linear regression models including CPP score, treatment group, and the CPP score × treatment group interaction were used to determine whether the associations differed among treatment conditions. For the fiber‐photometry analysis, each mouse contributed one CPP score and one corresponding Δ*AUC* value. Individual compartment‐entry events were summarized within each mouse and were not treated as independent biological observations. Because only three mice were included per group, the group‐specific Pearson coefficients, *P* values, and regression lines were reported for transparency but were interpreted strictly as descriptive and exploratory findings rather than definitive within‐group evidence. Ordinary least‐squares regression lines were fitted for visualization. Each data point represented one biologically independent mouse, and all statistical tests were two‐sided.

For the liposome‐distribution analysis, pooled and formulation‐stratified Pearson correlations were performed using matched CPP scores and VTA Coumarin 6 MFI values from the same mice in the DSPE‐mPEG‐HE and DSPE‐mPEG‐RVG29‐HE groups; because only four animals were available per formulation group, the stratified analyses were considered exploratory.

## Author Contributions

H.J. conceived and designed the study, performed the majority of the experiments, conducted data analysis, and drafted the original manuscript. Z.Z. carried out animal experiments and biochemical assays, performed formal analyses, and contributed to manuscript writing and revision. N.X. participated in experimental investigations and data analysis and contributed to manuscript revision. J.D., Y.Y.Z., X.L., and Y.X.W. assisted with animal experiments, molecular biology assays, and data acquisition. Y.Z. and G.X. contributed to data analysis and visualization. Y.T. and M.L. provided technical support and assisted with methodology optimization. Q.W. contributed to experimental design and data interpretation. H.M., Q.W., and Z.L. supervised the project, acquired funding, provided resources, and critically revised the manuscript. H.M., Q.W., and Z.L. are the corresponding authors and oversaw the overall direction of the study. All authors discussed the results and approved the final version of the manuscript.

## Conflicts of Interest

The authors declare no conflicts of interest.

## Supporting information




**Supporting File**: advs77820‐sup‐0001‐SuppMat.docx.

## Data Availability

The data that support the findings of this study are available from the corresponding author upon reasonable request.
