# Supplementary material for: Hederagenin Attenuates Morphine Addiction via GABAA1‐Mediated Modulation of Ca2 +–CaMK–CREB Signaling in Ventral Tegmental Dopaminergic Neurons
Source: Adv Sci (Weinh). 2026 Sep 27:e77820. Online ahead of print. doi: 10.1002/advs.77820 (PMC13616273; doi:10.1002/advs.77820)
Supplement: Supplementary file 1 [file ADVS-9999-e77820-s001.docx]

A

B

**Figure S1. Raw compartment occupancy during CPP baseline and test sessions.**Raw time spent in the drug-paired（WHITE), non-drug-paired (BLACK), and neutral compartments (MIDDLE) during the baseline(pretest) and test sessions of the CPP experiment.**(A)** The table shows the original compartment occupancy data for each group at baseline and test. **(B)**The neutral-compartment occupancy was further compared between the baseline(pretest) and test sessions to determine whether changes in CPP scores were confounded by altered time spent in the neutral compartment. Data are presented as mean ± SEM. Statistical analysis for neutral - compartment occupancy between baseline(pretest) and test was performed using a paired two-tailed Student’s t-test. ns, not significant.

A

**Figure S2.** **Sex-balanced behavioral validation of the inhibitory effect of hederagenin on morphine-induced CPP acquisition.**Pretest and test CPP scores from the sex-balanced validation experiment in male and female mice. Male and female mice were assigned to the Con, Mor, Mor + HE-LD, and Mor + HE-HD groups. HE was administered during the conditioning phase at 20 mg/kg (LD) or 80 mg/kg (HD) by oral gavage, and morphine was administered at 10 mg/kg, i.p. during morphine-paired conditioning sessions. In both sexes, morphine conditioning induced a significant increase in CPP scores, whereas HE treatment attenuated morphine-induced CPP acquisition. Data are presented as mean ± SEM. Male and female pretest and test data were analyzed separately using ordinary one-way ANOVA followed by Dunnett’s multiple-comparisons test, with the Mor group as the reference. **P < 0.01, ***P < 0.001, ****P < 0.0001.

A

**Figure S3. Docking protocol validation using a reference ligand.** Left, overall structure of the GABAA receptor used for docking, with the selected binding pocket highlighted. Middle, enlarged view of the docked reference ligand in the binding pocket, showing a representative interaction with ARG-173 at approximately 2.9 Å. Right, two-dimensional interaction diagram showing predicted non-covalent interactions between the reference ligand and surrounding residues. This validation was performed to support the suitability of the selected docking pocket and parameters before HE docking.

**Figure S4. Pooled and treatment-stratified associations between CPP scores, activation of VTA dopaminergic neurons, and VTA CREB signaling.**
**(A)** Pooled correlation between conditioned place preference (CPP) scores and the number of TH⁺/c-Fos⁺ double-positive cells in the ventral tegmental area (VTA) across the Con, Mor, and Mor + HE groups (Pearson r = 0.661, P = 0.0073; n = 15). **(B)** Treatment-stratified correlations between CPP scores and VTA TH⁺/c-Fos⁺ cell counts. The correlations were negative in the Con group (r = −0.992, P = 0.0009), positive in the Mor group (r = 0.997, P = 0.0002), and negative in the Mor + HE group (r = −0.989, P = 0.0014; n = 5 per group). An exploratory linear-model analysis showed a significant CPP score × treatment group interaction [*F*(2, 9) = 314.24, *P* < 0.0001, partial η² = 0.986], indicating that the direction and regression slope of the relationship differed across treatment groups. **(C)** Pooled correlation between CPP scores and the VTA p-CREB/CREB ratio (Pearson r = 0.900, P < 0.0001; n = 15). **(D)** Treatment-stratified correlations between CPP scores and the VTA p-CREB/CREB ratio. Positive correlations were observed in the Con group (r = 0.928, P = 0.0231) and Mor group (r = 0.995, P = 0.0005), whereas a positive but non-significant trend was observed in the Mor + HE group (r = 0.850, P = 0.0679; n = 5 per group). CPP score was calculated as the time spent in the morphine-paired compartment minus the time spent in the non-morphine-paired compartment during the CPP test. Each point represents one biologically independent mouse with individually matched behavioral and molecular data. Different colors and symbols indicate the Con, Mor, and Mor + HE groups. Solid lines represent ordinary least-squares regression fits. Pearson correlation coefficients and two-sided P values are shown. For each molecular endpoint, the correlation dataset included five mice per group: the three used for primary analysis in Figure 3 (TH⁺/c-Fos⁺) or Figure 7 (p-CREB/CREB), plus two additional mice with matched behavioral and molecular data.

**Figure S5. Animal-level association between CPP score and compartment-selective VTA calcium activity in a matched fiber-photometry subset of the overall CPP cohort.**

**(A)** Pooled correlation between CPP score and the compartment-selective calcium-response index in the Con, Mor, and Mor + HE groups. CPP score was calculated as the time spent in the drug-paired compartment minus that in the non-drug-paired compartment during the CPP test session. For each mouse, calcium activity was quantified as the area under the event-aligned ΔF/F curve during the 0–10 s interval following compartment entry. The compartment-selective calcium-response index was calculated as ΔAUC = AUC(drug-paired) − AUC(non-drug-paired). Each point represents one biologically independent mouse for which behavioral and fiber-photometry data were obtained from the same animal (n = 3 mice per group; n = 9 in total). The solid line represents the ordinary least-squares regression fitted to the pooled matched dataset. A significant positive correlation was observed between CPP score and ΔAUC in the pooled analysis (two-sided Pearson correlation, r = 0.883, P = 0.0016).**(B)** Group-specific regression visualization of the same matched dataset. Separate ordinary least-squares regression lines are shown for the Con, Mor, and Mor + HE groups. The regression slopes were positive in all three groups (Con: r = 0.996, P = 0.0602; Mor: r = 0.811, P = 0.398; Mor + HE: r = 0.988, P = 0.099). Because only three biologically independent mice were included in each group, these analyses are presented solely as descriptive and exploratory visualizations and were not used for definitive within-group statistical inference.

**Figure S6. Correlation analysis between CPP scores and VTA Coumarin 6 mean fluorescence intensity.** **(A)** Pooled Pearson correlation analysis between CPP scores and VTA Coumarin 6 mean fluorescence intensity (MFI) across all animals with matched behavioral and fluorescence data. CPP scores were significantly negatively correlated with VTA Coumarin 6 MFI (Pearson r = −0.979, P < 0.0001; n = 8 mice). CPP score was calculated as the time spent in the drug-paired compartment minus that in the non-drug-paired compartment during the post-test. **(B)** Formulation-stratified Pearson correlation analyses between CPP scores and VTA Coumarin 6 MFI. Negative correlation trends were observed in the mPEG-HE group (Pearson r = −0.929, P = 0.071; n = 4 mice) and the mPEG-RVG29-HE group (Pearson r = −0.969, P = 0.031; n = 4 mice). Blue circles indicate the mPEG-HE group, and green squares indicate the mPEG-RVG29-HE group. Each point represents one biologically independent mouse. Solid lines indicate ordinary least-squares linear regression fits.

**Table S1. Primer sequences.**

| Gene name | Primer type | Primer sequences (5’-3’) |
| --- | --- | --- |
| Camk2a | Forward | ACCTGCACCCGATTCACAG |
|  | Reverse | TGGCAGCATACTCCTGACCA |
| Camk2b | Forward | AGAGATCCGGGGCTTTATTGA |
|  | Reverse | AATGCTCTTTAGCTTGGAGCG |
| Camk2d | Forward | CAGCAGACGCCCTCCTAAAAG |
|  | Reverse | GGGTTGTAGATCCTGGGCTATC |
| Camk2g | Forward | TGATGTCCTGAGCTTGGTGAG |
|  | Reverse | GGGGGCTAATGGGAACTGG |
| Gabra1 | Forward | TGGTTGCCCTCATTGATGTCT |
|  | Reverse | CCCATCCCCATCTTCGTCC |
| GAPDH | Forward | AGGTCGGTGTGAACGGATTTG |
|  | Reverse | TGTAGACCATGTAGTTGAGGTCA |

**Table S2. Concise summary of key inferential statistics**

| **Figure / panel** | **Outcome and sample size** | **Statistical analysis** | **Main inferential results and key comparisons** |
| --- | --- | --- | --- |
| **Figure 1F–I** | cAMP AUC screening F: hederagenin; G: cauloside; H: oleanolic acid; I: magnoflorine F n=5–6/group; G n=5–7/group; H n=5–7/group; I n=5–6/group | Ordinary one-way ANOVA F–I: Dunnett (Mor reference) | F: *F*(4, 24) = 16.46, *P* < 0.0001, η² = 0.733 G: *F*(4, 27) = 19.24, *P* < 0.0001, η² = 0.740 H: *F*(4, 26) = 29.72, *P* < 0.0001, η² = 0.821  I: *F*(4, 22) = 16.98, *P* < 0.0001, η² = 0.755 F: Mor vs HE 8, 16, 32 μM, adjusted *P* = 0.0109, 0.0001, <0.0001. G: Mor vs cauloside C 8, 16, 32 μM, *P* = 0.0011, <0.0001, <0.0001. H: Mor vs oleanolic acid 8, 16, 32 μM, all *P* < 0.0001.  I: Mor vs magnoflorine 32 μM, *P* < 0.0001; 8 and 16 μM, ns. |
| **Figure 1J** | Baseline black- vs white-compartment time n = 48 paired mice | Paired two-tailed Student’s t-test | *t*(47) = 19.05, *P* < 0.0001, Cohen's *d*_z_ = 2.75; mean paired difference = 449.6 s (95% CI 402.1–497.1) Black-compartment time was greater than white-compartment time. |
| **Figure 1M** | CPP acquisition: pretest and test Pretest N=48; test N=48; n=8/group | Ordinary one-way ANOVA + Dunnett (Mor reference); phases analyzed separately | Pretest: *F*(5, 42) = 0.03, *P* = 0.9996, η² = 0.003 Test: *F*(5, 42) = 10.28, *P* < 0.0001, η² = 0.550 Pretest: all adjusted *P* > 0.999. Test: Mor vs Con *P* < 0.0001; vs LD *P* = 0.0022; vs MD, HD, and Jitai all *P* < 0.0001. |
| **Figure 1N** | Established CPP expression in male and female cohorts n = 6/group for each sex | Pretests and female test: ordinary one-way ANOVA + Dunnett. Male test: Welch one-way ANOVA + Dunnett’s T3. | Male pretest: *F*(2, 15) = 2.16, *P* = 0.1503, η² = 0.223 Male test: *W*(2, 9.645) = 13.40, *P* = 0.0016, η² (descriptive) = 0.664 Female pretest: *F*(2, 15) = 1.11, *P* = 0.3562, η² = 0.129 Female test: *F*(2, 15) = 16.84, *P* = 0.0001, η² = 0.692 Male test: Mor vs Con *P* = 0.0009; Mor vs Expression *P* = 0.0044. Female test: Mor vs Con *P* = 0.0001; Mor vs Expression *P* = 0.0024. Both pretests: all planned comparisons ns. |
| **Figure S2A** | CPP acquisition under matched conditioning-time conditions in male and female cohorts n = 6/group for each sex | Male and female pretest and test data were analyzed separately using ordinary one-way ANOVA + Dunnett (Mor reference). | Male pretest: *F*(3, 20) = 2.07, *P* = 0.1363, η² = 0.237; all Dunnett-adjusted comparisons versus Mor were ns. Male test: *F*(3, 20) = 13.59, *P* < 0.0001, η² = 0.671; Mor vs Con *P* < 0.0001, Mor vs LD *P* = 0.0036, and Mor vs HD *P* < 0.0001. Female pretest: *F*(3, 20) = 0.25, *P* = 0.8624, η² = 0.036; all Dunnett-adjusted comparisons versus Mor were ns. Female test: *F*(3, 20) = 12.47, *P* < 0.0001, η² = 0.652; Mor vs Con *P* = 0.0002, Mor vs LD *P* = 0.0021, and Mor vs HD *P* < 0.0001. |
| **Figure 2B** | Sucrose preference test: total liquid intake and sucrose preference at 12 and 24 h n = 5/group | Ordinary one-way ANOVA + Tukey | Intake 12 h: *F*(3, 16) = 2.60, *P* = 0.0884, η² = 0.327 Intake 24 h: *F*(3, 16) = 1.30, *P* = 0.3094, η² = 0.196 Sucrose 12 h: *F*(3, 16) = 0.46, *P* = 0.7171, η² = 0.079 Sucrose 24 h: *F*(3, 16) = 0.75, *P* = 0.5389, η² = 0.123 All Tukey-adjusted comparisons were nonsignificant. |
| **Figure 2C** | Elevated plus maze: zone times and entries n = 5/group | C1–C4: ordinary one-way ANOVA + Tukey. C5–C6: Welch one-way ANOVA + Dunnett’s T3 (Con reference). | Center time: *F*(3, 16) = 1.23, *P* = 0.3307, η² = 0.188 Open-arm time: *F*(3, 16) = 2.26, *P* = 0.1211, η² = 0.297 Closed-arm time: *F*(3, 16) = 2.91, *P* = 0.0664, η² = 0.353 Center entries: *F*(3, 16) = 1.54, *P* = 0.2435, η² = 0.224 Open-arm entries: *W*(3, 7.221) = 1.40, *P* = 0.3190, η² (descriptive) = 0.244 Closed-arm entries: *W*(3, 8.1) = 1.47, *P* = 0.2944, η² (descriptive) = 0.167 All multiplicity-adjusted comparisons were nonsignificant. |
| **Figure 2D** | Open-field test: center entries, total distance, and activity time n = 5/group | Center entries and activity time: ordinary one-way ANOVA + Tukey. Total distance: Welch one-way ANOVA + Dunnett’s T3. | Center entries: *F*(3, 16) = 0.20, *P* = 0.8978, η² = 0.035 Total distance: *W*(3, 6.971) = 1.48, *P* = 0.3010, η² (descriptive) = 0.149 Activity time: *F*(3, 16) = 0.26, *P* = 0.8515, η² = 0.047 All multiplicity-adjusted comparisons were nonsignificant. |
| **Figure 2E — latency** | Morris water maze: latency to platform on Days 1–4 and test n = 5/group at each time point | Separate ordinary one-way ANOVA + Tukey at each time point | Day 1: *F*(3, 16) = 0.53, *P* = 0.6699, η² = 0.090 Day 2: *F*(3, 16) = 1.40, *P* = 0.2803, η² = 0.207 Day 3: *F*(3, 16) = 1.46, *P* = 0.2632, η² = 0.215 Day 4: *F*(3, 16) = 0.74, *P* = 0.5441, η² = 0.122 Test: *F*(3, 16) = 1.12, *P* = 0.3710, η² = 0.173 All Tukey-adjusted comparisons were nonsignificant. |
| **Figure 2E — distance** | Morris water maze: distance to platform on Days 1–4 and test n = 5/group at each time point | Day 1: Welch one-way ANOVA. Days 2–4 and test: ordinary one-way ANOVA + Tukey. | Day 1: *W*(3, 7.933) = 0.58, *P* = 0.6423, η² (descriptive) = 0.106 Day 2: *F*(3, 16) = 1.85, *P* = 0.1786, η² = 0.258 Day 3: *F*(3, 16) = 2.00, *P* = 0.1544, η² = 0.273 Day 4: *F*(3, 16) = 2.30, *P* = 0.1162, η² = 0.301 Test: *F*(3, 16) = 1.68, *P* = 0.2104, η² = 0.240 All multiplicity-adjusted comparisons were nonsignificant. Day 4 uses the corrected LD values. |
| **Figure 2E — speed** | Morris water maze: swimming speed on Days 1–4 and test n = 5/group at each time point | Separate ordinary one-way ANOVA + Tukey at each time point | Day 1: *F*(3, 16) = 0.89, *P* = 0.4688, η² = 0.143 Day 2: *F*(3, 16) = 1.88, *P* = 0.1729, η² = 0.261 Day 3: *F*(3, 16) = 1.45, *P* = 0.2656, η² = 0.214 Day 4: *F*(3, 16) = 2.22, *P* = 0.1255, η² = 0.294 Test: *F*(3, 16) = 0.88, *P* = 0.4715, η² = 0.142 All Tukey-adjusted comparisons were nonsignificant. |
| **Figure 3E** | c-Fos-positive cell counts in VTA, NAc, and hippocampus n = 3 mice/group | Separate ordinary one-way ANOVA + Dunnett (Mor reference) | VTA: *F*(2, 6) = 11.82, *P* = 0.0083, η² = 0.798 NAc: *F*(2, 6) = 14.27, *P* = 0.0052, η² = 0.826 Hip: *F*(2, 6) = 0.03, *P* = 0.9727, η² = 0.009 VTA: Mor vs Con *P* = 0.0074; Mor vs HE *P* = 0.0151. NAc: Mor vs Con *P* = 0.0057; Mor vs HE *P* = 0.9553. Hip: both comparisons ns. |
| **Figure 3F** | VTA TH+/c-Fos+ double-positive cells n = 3 mice/group | Ordinary one-way ANOVA + Dunnett (Mor reference) | *F*(2, 6) = 34.93, *P* = 0.0005, η² = 0.921 Mor vs Con *P* = 0.0003; Mor vs HE *P* = 0.0023. |
| **Figure 6F** | VTA c-Fos after GABAA1 knockdown n = 3 mice/group | Ordinary one-way ANOVA + Dunnett (Mor reference) | *F*(4, 10) = 10.13, *P* = 0.0015, η² = 0.802 Mor vs Con *P* = 0.0029; Mor vs LD, MD, and HD all ns. |
| **Figure 6G** | CPP score after GABAA1 knockdown: pretest and test n = 6/group | Separate ordinary one-way ANOVA + Dunnett (Mor reference) | Pretest: *F*(4, 25) = 1.28, *P* = 0.3044, η² = 0.170 Test: *F*(4, 25) = 28.18, *P* < 0.0001, η² = 0.818 Pretest: all comparisons ns. Test: Mor vs Con *P* < 0.0001; Mor vs LD, MD, and HD all ns. |
| **Figure 7A** | Intracellular Ca^2+^ after morphine with graded HE n=11–12/group; N=70 | Ordinary one-way ANOVA + Dunnett (Mor reference) | *F*(5, 64) = 3.52, *P* = 0.0072, η² = 0.215 Mor vs Con *P* = 0.0058; Mor vs HE 0.1, 1, 10, and 32 μM: *P* = 0.2630, 0.0407, 0.0174, and 0.0026. |
| **Figure 7B** | Basal intracellular Ca^2+^ after HE alone n=9–11/group; N=58 | Ordinary one-way ANOVA + planned Dunnett comparisons | *F*(5, 52) = 14.85, *P* < 0.0001, η² = 0.588 Mor vs Con *P* < 0.0001; Con vs HE-alone 32 μM *P* > 0.9999. The HE-alone conclusion is based on comparisons with Con. |
| **Figure 7C** | CCK-8 cell viability n=3/group; N=30 | Ordinary one-way ANOVA + Tukey | *F*(9, 20) = 1.09, *P* = 0.4121, η² = 0.329 All Tukey-adjusted comparisons were nonsignificant. |
| **Figure 7D** | VTA CaMK2-family mRNA CAMK2A n=8–9/group; CAMK2B/D/G n=9/group | Separate ordinary one-way ANOVA + Tukey | CAMK2A: *F*(2, 22) = 16.42, *P* < 0.0001, η² = 0.599 CAMK2B: *F*(2, 24) = 1.27, *P* = 0.3000, η² = 0.095 CAMK2D: *F*(2, 24) = 2.77, *P* = 0.0829, η² = 0.187 CAMK2G: *F*(2, 24) = 0.76, *P* = 0.4780, η² = 0.060 CAMK2A: Con vs Mor *P* = 0.000096; Mor vs HE *P* = 0.000268; Con vs HE *P* = 0.8342. Other genes: all pairwise comparisons ns. |
| **Figure 7E** | NAc CaMK2-family mRNA n=8–9/group, depending on gene | Separate ordinary one-way ANOVA + Tukey | CAMK2A: *F*(2, 23) = 2.92, *P* = 0.0743, η² = 0.202 CAMK2B: *F*(2, 24) = 1.81, *P* = 0.1855, η² = 0.131 CAMK2D: *F*(2, 21) = 3.05, *P* = 0.0687, η² = 0.225 CAMK2G: *F*(2, 23) = 0.03, *P* = 0.9704, η² = 0.003 All Tukey-adjusted comparisons were nonsignificant. |
| **Figure 7F** | mPFC CaMK2-family mRNA n = 9/group | Separate ordinary one-way ANOVA + Tukey | CAMK2A: *F*(2, 24) = 0.00, *P* = 0.9988, η² = 0.000 CAMK2B: *F*(2, 24) = 1.78, *P* = 0.1902, η² = 0.129 CAMK2D: *F*(2, 24) = 0.39, *P* = 0.6845, η² = 0.031 CAMK2G: *F*(2, 24) = 0.17, *P* = 0.8483, η² = 0.014 All Tukey-adjusted comparisons were nonsignificant. |
| **Figure 7H** | VTA phosphorylation ratios: p-CaMKII/CaMKII, p-PKA/PKA, p-ERK/ERK, and p-CREB/CREB n = 3/group | Separate ordinary one-way ANOVA + Dunnett (Mor reference) | p-CaMKII/CaMKII: *F*(2, 6) = 46.51, *P* = 0.0002, η² = 0.939 p-PKA/PKA: *F*(2, 6) = 54.67, *P* = 0.0001, η² = 0.948 p-ERK/ERK: *F*(2, 6) = 42.63, *P* = 0.0003, η² = 0.934 p-CREB/CREB: *F*(2, 6) = 38.05, *P* = 0.0004, η² = 0.927 Mor vs Con: *P* = 0.0001, 0.0001, 0.0003, and 0.0002. Mor vs HE: *P* = 0.0166, 0.0003, 0.0004, and 0.0080, respectively. |
| **Figure 9C** | Serial in vivo fluorescence at 2, 4, 8, and 12 h n = 3/group/time point | Separate ordinary one-way ANOVA + Tukey at each time point | 2 h: *F*(2, 6) = 0.22, *P* = 0.8092, η² = 0.068 4 h: *F*(2, 6) = 9.13, *P* = 0.0151, η² = 0.753 8 h: *F*(2, 6) = 62.25, *P* = 0.000097, η² = 0.954 12 h: *F*(2, 6) = 58.34, *P* = 0.000117, η² = 0.951 2 h: all ns. 4 h: mPEG vs RVG29 *P* = 0.0130. 8 h: mPEG vs RVG29 *P* = 0.000103. 12 h: mPEG vs RVG29 *P* = 0.000116; HE-loaded mPEG vs RVG29 *P* = 0.000577. |
| **Figure 9F** | CPP expression cohort: pretest and test n = 8/group | Pretest and test: Welch’s one-way ANOVA + Dunnett’s T3 (Mor reference). | Pretest: W(4, 16.97) = 0.7630, P = 0.5637, η² (descriptive) = 0.094.  Test: W(4, 16.48) = 65.63, P < 0.0001, η² (descriptive) = 0.889. Pretest: all comparisons ns. Test: Mor vs Con, DSPE-mPEG-HE, DSPE-mPEG-RVG29-HE, and free HE: all adjusted *P* < 0.0001. |
| **Figure S4A–B** | CPP score vs VTA TH^+^/c-Fos^+^ cells; treatment interaction Pooled matched animals; interaction df as shown  *n* = 15 matched mice | Two-sided Pearson correlation; linear-model interaction F-test | Pooled correlation: *r* = 0.661, *P* = 0.0073 CPP × treatment interaction: *F*(2, 9) = 314.24, *P* < 0.0001, partial η² = 0.986 Treatment-stratified regression slopes were interpreted with the interaction test. |
| **Figure S4C–D** | CPP score vs VTA p-CREB/CREB; treatment interaction Pooled matched animals; interaction df as shown  *n* = 15 matched mice | Two-sided Pearson correlation; linear-model interaction F-test | Pooled correlation: *r* = 0.900, *P* < 0.0001 CPP × treatment interaction: *F*(2, 9) = 113.47, *P* < 0.0001, partial η² = 0.962 Treatment-stratified regression slopes were interpreted with the interaction test. |
| **Figures S5A–B and S6A–B** | Animal-level associations of CPP score with compartment-selective VTA calcium activity and VTA Coumarin 6 MFI.  Figure S5A–B: CPP score versus VTA calcium ΔAUC; n = 9 matched mice in total, including Con, Mor, and Mor + HE groups (n = 3 per group).  Figure S6A–B: Post-test CPP score versus VTA Coumarin 6 MFI; n = 8 matched mice in total, including DSPE-mPEG-HE and DSPE-mPEG-RVG29-HE groups (n = 4 per group). | Two-sided Pearson correlation analyses; ordinary least-squares regression lines were fitted for visualization. Pooled and group- or formulation-stratified analyses were performed. | S5A–B, CPP vs VTA calcium ΔAUC: pooled *r* = 0.883, *P* = 0.0016, *n* = 9; Con: *r* = 0.996, *P* = 0.0602; Mor: *r* = 0.811, P = 0.3980; Mor + HE: r = 0.988, P = 0.099 (n = 3/group; group-specific analyses descriptive and exploratory).  S6A–B, post-test CPP vs VTA Coumarin 6 MFI: pooled r = −0.979, P < 0.0001, n = 8; DSPE-mPEG-HE: r = −0.929, P = 0.071; DSPE-mPEG-RVG29-HE: r = −0.969, P = 0.031 (n = 4/group; within-formulation analyses were considered descriptive and exploratory). |
